# A Conserved Oct4/POUV-Dependent Network Links Adhesion and Migration to Progenitor Maintenance

**DOI:** 10.1016/j.cub.2013.09.048

**Published:** 2013-11-18

**Authors:** Alessandra Livigni, Hanna Peradziryi, Alexei A. Sharov, Gloryn Chia, Fella Hammachi, Rosa Portero Migueles, Woranop Sukparangsi, Salvatore Pernagallo, Mark Bradley, Jennifer Nichols, Minoru S.H. Ko, Joshua M. Brickman

**Affiliations:** 1 MRC Centre for Regenerative Medicine, Institute for Stem Cell Research, School of Biological Sciences, 5 Little France Drive, University of Edinburgh, Edinburgh EH16 4UU, UK; 2 The Danish Stem Cell Centre (DanStem), University of Copenhagen, 3B Blegdamsvej, 2200 Copenhagen, Denmark; 3 Laboratory of Genetics, National Institute on Aging, NIH Biomedical Research Centre, 251 Bayview Boulevard, Suite 100, Baltimore, MD 21224, USA; 4 Wellcome Trust Centre for Stem Cell Research, University of Cambridge, Tennis Court Road, Cambridge CB2 1QR, UK; 5 School of Chemistry, Joseph Black Building, King’s Buildings, the University of Edinburgh, West Mains Road, Edinburgh EH9 3JJ, UK; 6 Department of Systems Medicine, Keio University School of Medicine, 35 Shinanomachi, Shinjuku-ku, Tokyo 160, Japan

## Abstract

**Background:**

The class V POU domain transcription factor Oct4 (Pou5f1) is a pivotal regulator of embryonic stem cell (ESC) self-renewal and reprogramming of somatic cells to induced pluripotent stem (iPS) cells. Oct4 is also an important evolutionarily conserved regulator of progenitor cell differentiation during embryonic development.

**Results:**

Here we examine the function of Oct4 homologs in *Xenopus* embryos and compare this to the role of Oct4 in maintaining mammalian embryo-derived stem cells. Based on a combination of expression profiling of Oct4/POUV-depleted *Xenopus* embryos and in silico analysis of existing mammalian Oct4 target data sets, we defined a set of evolutionary-conserved Oct4/POUV targets. Most of these targets were regulators of cell adhesion. This is consistent with Oct4/POUV phenotypes observed in the adherens junctions in *Xenopus* ectoderm, mouse embryonic, and epiblast stem cells. A number of these targets could rescue both Oct4/POUV phenotypes in cellular adhesion and multipotent progenitor cell maintenance, whereas expression of cadherins on their own could only transiently support adhesion and block differentiation in both ESC and *Xenopus* embryos.

**Conclusions:**

Currently, the list of Oct4 transcriptional targets contains thousands of genes. Using evolutionary conservation, we identified a core set of functionally relevant factors that linked the maintenance of adhesion to Oct4/POUV. We found that the regulation of adhesion by the Oct4/POUV network occurred at both transcriptional and posttranslational levels and was required for pluripotency.

## Highlights

• Evolutionary conservation reduces the complexity of Oct4 targets
• Conserved Oct4 targets are regulators of adhesion in addition to differentiation
• Adhesion proteins such as E-cadherin transiently rescue Oct4 phenotypes
• Transcription factor targets of Oct4 rescue sustained adhesion and differentiation

## Graphical Abstract

**Figure:**
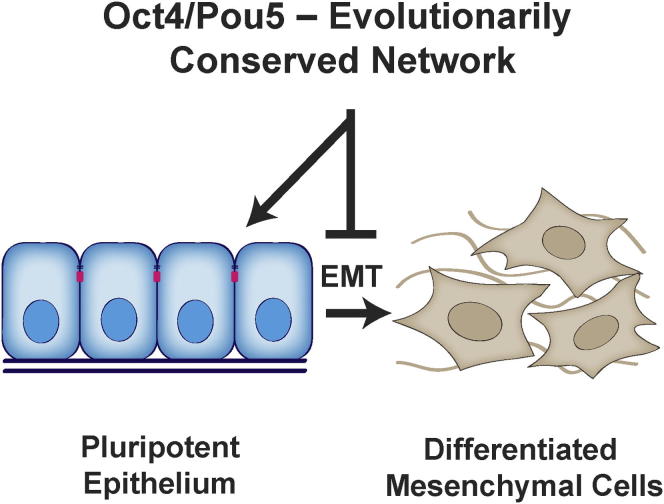

## Introduction

In vertebrate development, lineage specification occurs progressively with time and utilizes pools of pluri- and multipotent progenitor cells with capacity to populate the different embryonic lineages. Naive embryonic stem cells (ESCs) and primed epiblast stem cells (EpiSCs) are pluripotent cell lines derived from early embryos that self-renew indefinitely in vitro [1]. The self-renewal of pluripotent cells is regulated by defined extrinsic signals and coordinated by a gene regulatory network featuring the Class V POU transcription factor Oct4 [2, 3]. Oct4 is also the central transcription factor in the reprogramming of somatic cells to induced pluripotent stem cells (iPSCs) [4, 5].

In vitro Oct4 has been shown to function as both an activator and repressor of gene transcription, but activation of Oct4 targets is sufficient to block differentiation and induce reprogramming [6]. Consequently, to understand the role of Oct4/POUV in maintaining pluripotency and supporting embryonic development, it is essential to decipher the function of the network activated by Oct4 and its homologs.

Currently, genome-wide chromatin occupancy studies indicate that Oct4 binds thousands of targets [7, 8]; however, only a fraction of these genes may be functionally relevant to pluripotency. Our approach to identifying the relevant subset of targets is to look for evolutionary conservation. Although Oct4 activity in mammals and naive ESCs is associated with preimplantation development, Oct4 is also expressed in gastrulation-stage progenitors and primed pluripotent cells such as EpiSCs and human ESCs. This expression is conserved in nonmammalian vertebrates where Oct4 homologs prevent premature differentiation of germ layer progenitors [9, 10, 11, 12]. These homologs, particularly the *Xenopus* POUV proteins, can support ESC self-renewal in the absence of Oct4 [9, 12] and induce pluripotency in the reprogramming of human and murine somatic cells [5]. Here we identify a set of conserved Oct4/POUV targets using a comparison of the *Xenopus* network to existing mammalian genome-wide data. Based on this data set and a series of gain- and loss-of-function experiments, we uncovered a novel role for Oct4/POUV in the maintenance of cell-cell adhesion that is essential for regulating differentiation.

## Results

### Identification of POUV-Regulated Genes in *Xenopus* Gastrulation

**Figure 1:**
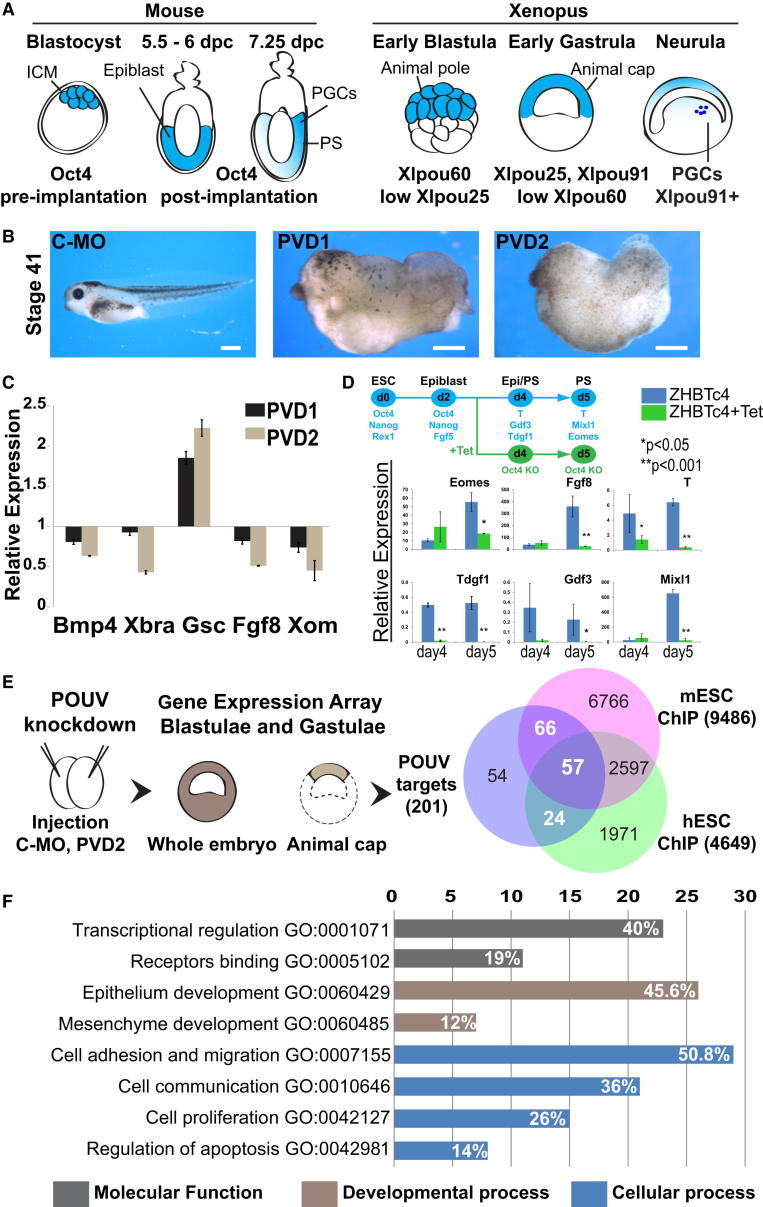
Identification of Conserved POUV Targets (A) Graphical representation of Oct4/POUV expression patterns in *M. musculus* and *X. laevis*. (B) POUV morphant phenotypes. Two-cell-stage embryos were injected with 120 ng of Control-MO or morpholino combinations; POUV-depleted (PVD), a mixture of Xlpou25, Xlpou60, and Xlpou91 MOs; and POUV-depleted-2 (PVD2), the same mixture but with a new Xlpou25 MO targeting both pseudoalleles of Xlpou25. Tadpoles were photographed at stage 41. The scale bar represents 0.5 mm. (C) qRT-PCR analysis of stage 10 embryos. Expression levels were normalized to *Odc* (ornithine decarboxylase) and are relative to the Control-MO. Data are shown as mean ± SD. (D) ZHBTc4 mESCs expressing Oct4 as a tetracycline (Tet)-suppressible transgene were differentiated to mesendoderm. Tet was added as indicated and qRT-PCR performed to monitor gene expression in differentiation. Expression is normalized to TBP and relative to ESCs. Data are shown as mean ± SD. p values were calculated by ANOVA test of minimum of four independent experiments. (E) POUV-dependent gene expression. Ectoderm (animal caps) from Control-MO and PVD2 embryos was dissected at early blastula stage (stage 8) and cultured until intact sibling embryos reached late blastulae (stage 9) and early gastrulae (stage 10) stages, as depicted. RNA from these explants was applied to Agilent 44K microarrays. POUV-regulated genes (N = 307) were identified by ANOVA analysis. Mammalian homologs (N = 201) of the POUV targets were identified by PSI-BLAST with minimum 60% identity. The Venn diagram shows the overlap of the genes regulated by POUV in *Xenopus* (N = 201) with global ChIP assays in murine and human ESCs. (F) Enriched Gene Ontologies of the targets conserved in mouse and human (N = 57) show the number of genes included in each GO term. White values on the bars show the percentage of genes included in the conserved targets list (N = 57). See also Figure S1 and Tables S1 and S2.

In *Xenopus laevis* there are three POUV genes, Xlpou25 (pou5f3.2), Xlpou60 (pou5f3.3), and Xlpou91 (pou5f3.1) [13, 14], whose expression pattern closely recapitulates the pre- and postimplantation expression of Oct4 in murine embryos (Figure 1A). Knockdown (KD) phenotypes for all the individual proteins have been described using different morpholino antisense oligos (MO) combinations [9, 11, 15, 16]. We found that our original depletion of POUV activity (PVD1, POUV-depleted 1; [9]) could be improved through the inclusion of additional MOs that take into account potential pseudoalleles (PVD2, POUV-depleted 2; Table S1 available online). We validated the absence of POUV activity in PVD2 using a synthetic POUV-responsive reporter gene (Figure S1A) and assessed the specificity of the new Xlpou25 MO by in vitro translation (Figure S1B) and rescue experiments (Figure S1C). Inclusion of the new MOs enhanced the penetrance and expressivity of the combinatorial KD (Figures 1B and 1C). Phenotypes for a single Xlpou91 morphant were described previously [9, 15]; with the new Xlpou25 MO we also observed a phenotype with similarities to that described by Cao et al. [11] (Table S1). PVD2 embryos exhibited a composite of these individual morphant phenotypes. Thus, PVD2 embryos displayed global axis extension defect, whereas Xlpou25 morphants appeared to have strong neural and Xlpou91 morphants a mesodermal convergent extension defect (Figure S1C). Additionally, we found that POUV proteins had distinct effects on gene expression (Figure S1D) and could regulate each other’s transcription (Figure S1E). We also observed that Oct4 depletion during mouse ESC differentiation toward epiblast and primitive streak [17], using the Tet-suppressible ZHBTc4 ESCs [18], led to similar changes in gene expression as PVD2 in *Xenopus* (Figure 1D; see diagrams in Figures 5A and 6A). Taken together, these observations argue a triple knockdown was essential for assessing the POUV downstream network in *Xenopus*.

To define the targets regulated by POUV proteins during germ layer formation we examined the transcriptional profile of PVD2 embryos and ectoderm (animal caps) at late blastula (stage 9) and early gastrula (stage 10) stages (Figure 1E). Using a three-way ANOVA test, we identified 182 downregulated genes, 45 upregulated genes, and 80 nonannotated genes (false discovery rate [FDR] < 0.05) that responded to POUV depletion (Table S2). Randomly selected genes were validated by quantitative RT-PCR (qRT-PCR) on independent samples (Figure S1F).

### Conservation of Oct4/POUV Target Genes

A number of studies have attempted to define Oct4 targets through genome-wide chromatin immunoprecipitation (ChIP) experiments. We assembled nonredundant lists of putative direct Oct4 targets (Table S2) described in murine ESCs (9,486 genes) [7, 8, 19, 20, 21, 22] and human ESCs (4,649 genes) [23, 24, 25]. The large size of these data sets may reflect the inherent limitations of genome-wide ChIP because it identifies DNA sites irrespective of functional significance. For Oct4 this is a problem because most RNA polymerase II promoters contain “octamer motifs” that are recognized by a broad family of POU proteins. We focused on the subset of these targets that is also regulated in *Xenopus* to identify conserved mediators of lineage specification. We identified 201 putative mammalian homologs from our POUV-responsive genes (N = 307) (Table S2). We found that 73% (N = 147) of our conserved homologs (N = 201) were contained within the combined Oct4 direct targets data set for either mouse or human and 28% (N = 57) overlapped with both mouse and human (Figure 1E; Table S2). This enrichment was highly significant (p = 4.8 × 10^−12^ for mESC overlap; p = 2.3 × 10^−13^ for hESC overlap) and constitutes a robust simplification of the Oct4 targets. Comparison of our profiling results to published expression data indicated that approximately 40% (N = 24 out of 57) of the genes conserved in mouse, human, and *Xenopus* responded to Oct4 knockdown in ESCs (Table S2) [20].

To further explore the functions of the conserved network we performed a Gene Ontology enrichment analysis [26], which revealed an abundance of regulators of epithelium development and cell adhesion among conserved POUV targets. Further annotation and literature mining revealed that 50.8% of the Oct4 targets conserved in *Xenopus*, mouse, and human (N = 29 out of 57) have been implicated in the regulation of cell adhesion and migration (Figure 1F; Table S2). This figure represents a dramatic enrichment (11-fold) over the occurrence of this gene class in the genome (p = 1.9 × 10^−15^).

### Oct4/POUV Proteins Control Cell Adhesion in Multipotent Ectoderm

**Figure 2:**
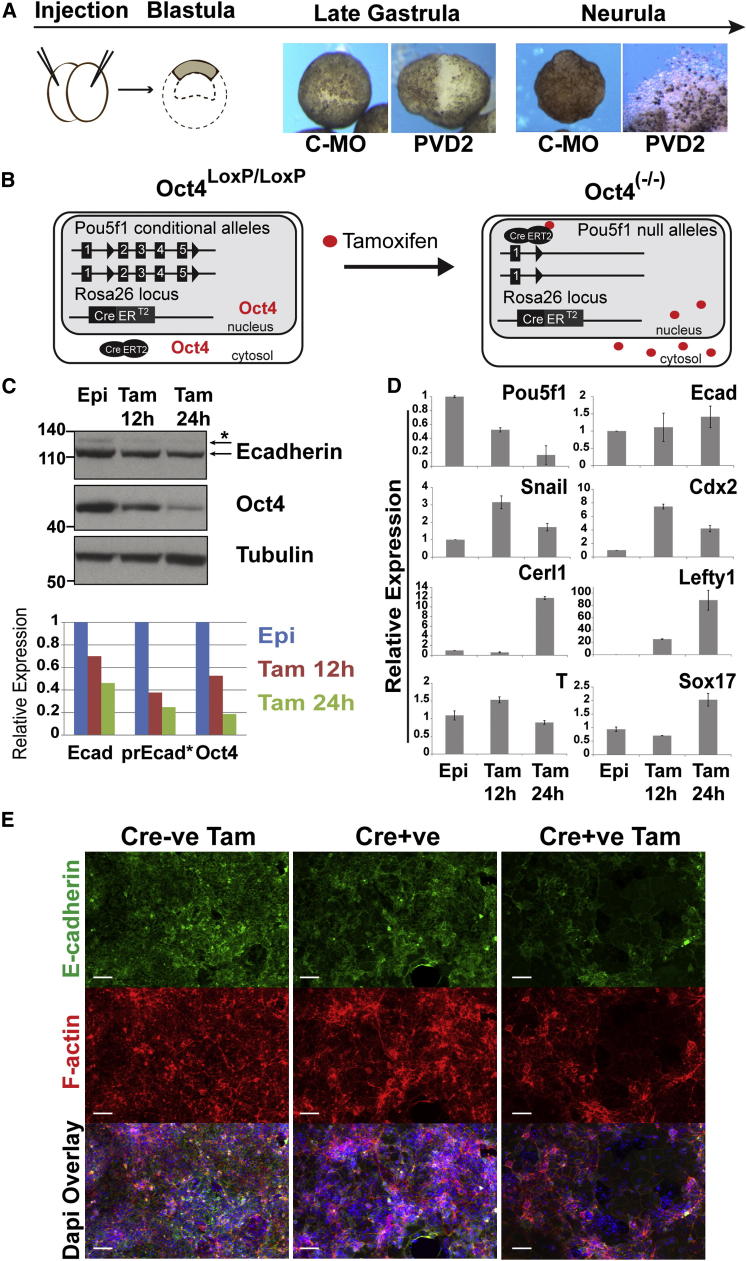
POUV Depletion Phenotypes in *Xenopus* Ectoderm and EpiSCs (A) POUV morphant ectodermal explants exhibit adhesion defects. Animal caps were excised from stage 8 blastulae injected with Control-MO or PVD2 and cultured until either late gastrula or neurula stage (stage 14). PVD2 morphant explants were slow to round up and never properly healed; by the completion of neurulation, PVD2 explants completely disaggregated. (B) Diagram of the *Oct4*^*LoxP/LoxP*^-EpiSC lines. These cells possess two conditional *Pou5f1* alleles and express an inducible Cre recombinase. Addition of tamoxifen induces Cre-mediated Oct4 deletion. (C) Immunoblot of samples from *Oct4*^*LoxP/LoxP*^ and *Oct4*^*(−/−)*^*-*EpiSC 12 and 24 hr after tamoxifen treatment (0.1 μM). The bottom panel shows quantified chemiluminescent signals normalized to tubulin and relative to untreated EpiSCs. Pr-Ecad is the precursor of E-cadherin, indicated by the starred arrow. (D) qRT-PCR of *Oct4*^*LoxP/LoxP*^-EpiSCs 12 and 24 hr after tamoxifen addition. Expression was normalized to TBP, relative to untreated EpiSCs. Bars show mean ± SD. (E) Immunofluorescence of Cre-negative (Cre-ve) and Cre-positive (Cre+ve) *Oct4*^*LoxP/LoxP*^-EpiSCs 48 hr after tamoxifen treatment. The scale bar represents 50 μm. See also Figure S2.

We examined the adhesive properties of POUV-depleted *Xenopus* ectoderm (animal caps, a multipotent tissue equivalent to the murine epiblast). PVD2 animal caps displayed defective healing in comparison to controls and lost their integrity when they reached neurula stage (stage 14) (Figure 2A). By performing single MO KDs, we observed a similar, although less severe, loss of adhesion with Xlpou25 KD, whereas the integrity of Xlpou60- or Xlpou91-depleted explants was not affected (Figure S2A). Xlpou25 is the most abundant POUV protein expressed during gastrulation [14], and these results suggest that it is required to maintain ectoderm integrity.

In *Xenopus*, the ectoderm consists of two layers: a monolayer epithelium of superficial cells (outer layer) and an inner layer of nonepithelial deep cells (sensorial layer). Cell adhesion of the superficial layer is maintained by tight junctions [27] and by the expression of C- and E-cadherin [28]. Inner cells are loosely attached to each other, and their adhesion is mediated by NF-protocadherin [29]. PVD2 inner cells were able to form normal aggregates with control outer layers, whereas PVD2 superficial cells lost their integrity when aggregated with control sensorial cells (Figure S2B), indicating a failure of tight junctions in the superficial layer of PVD2 morphants. Defective epiboly, observed in PVD2 embryos, also suggests a failure in ectodermal adhesion. PVD2 morphant gastrulae also displayed thickened ectoderm and a collapsed blastocoel containing enlarged blastomeres (Figure S2C). These ectodermal phenotypes closely resemble those obtained by depletion of C-cadherin [30] or overexpression of a dominant negative E-cadherin [31].

One possible explanation for this phenotype was that the loss of adhesion is a result of progressive cell death stemming from POUV depletion, and we evaluated the effect of PVD2 on cell viability. Control and PVD2 MOs showed similar degrees of cell death in ectoderm explants treated with Sytox Green (Figure S2D) and in TUNEL-stained embryos (Figure S2E). This suggests that loss of adhesion is not a consequence of cell death in PVD2 morphants, similar to adhesion phenotypes observed as a result of inhibition of NF-protocadherin [29], activation of EphrinB1 signaling [32], EphA4 signaling [33], or expression of a Xlim5-Engrailed fusion protein [34].

To test whether Oct4/POUV factors also regulate cell adhesion in mammalian differentiation, we used a conditional Oct4 mutant EpiSC line (*Oct4*^*LoxP/LoxP*^) derived from E6.5 epiblasts of mice homozygous for an Oct4 conditional allele. This line has LoxP sites inserted between exon 2 and exon 5 of the *Pou5f1* gene. It also harbors a tamoxifen-dependent Cre recombinase in the Rosa26 locus (Figure 2B), enabling tamoxifen-induced depletion of Oct4 as a result of Cre-mediated deletion of both *Oct4* alleles [*Oct4*^*(−/−)*^; Figure 2B]. We confirmed that *Oct4* transcript was lost and protein reduced to 10% of wild-type levels within 24 hr of Cre induction (Figures 2C and 2D). We then assessed the epithelialization and differentiation of these cultures. Induced *Oct4*^*(−/−)*^ EpiSCs displayed partial loss of adherens junctions, reduction of E-cadherin membrane staining and F-actin polymerization (Figure 2E), and differentiation toward endoderm (Figure 2D). Total E-cadherin protein was decreased (Figure 2C); however, we observed no reduction in *E-cadherin* transcript, despite a transient increase in *Snail* transcription, suggesting that E-cadherin regulation was posttranscriptional (Figure 2D). Taken together, the data in both *Xenopus* and mouse suggest that the Oct4/POUV network in gastrulation-stage progenitors acts to stabilize adhesion through the regulation of E-cadherin at the cell membrane.

### Conserved POUV Targets Rescue Adhesion Phenotypes

**Figure 3:**
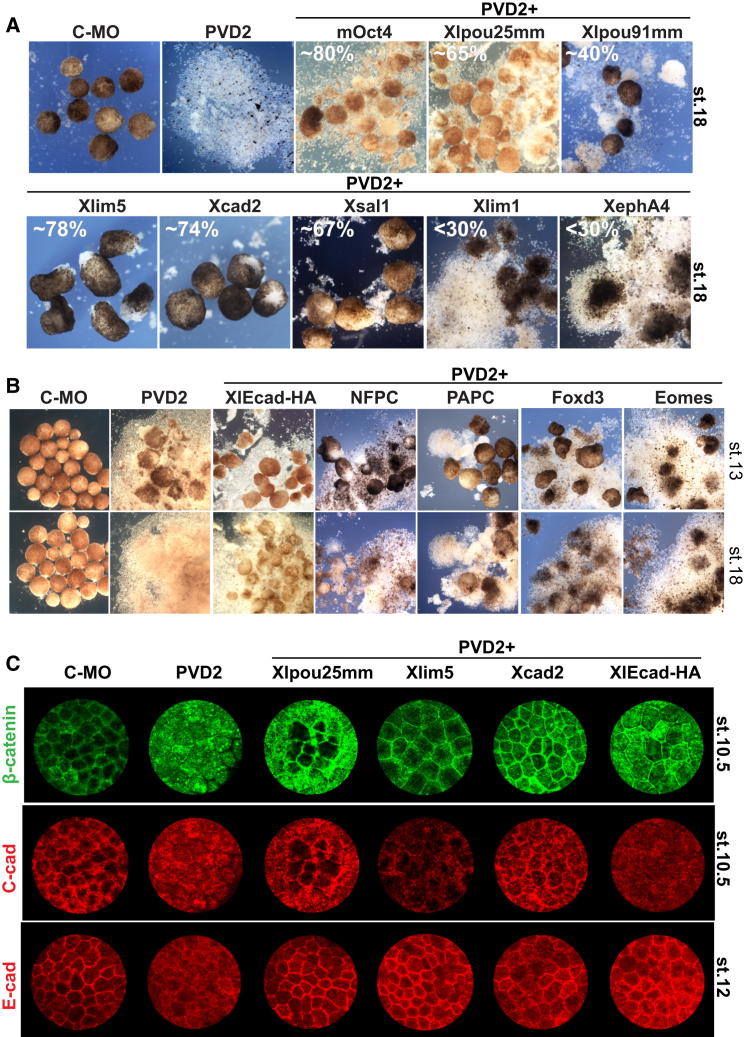
Rescue of Adhesion Phenotypes by POUV Targets (A) Rescue of ectoderm adhesion. Embryos were injected with 120 ng of Control-MO or PVD2 and 500 pg of mRNA encoding the indicated targets (1 ng for mOct4). Animal caps were cultured to neurula stage (stage 14). PVD2 animal caps displayed a dramatic loss of cell adhesion, which was rescued by murine Oct4 and by some of the conserved targets. Rescue index is reported as percentage of intact animal caps, and further details are summarized in Table S3. (B) Rescue of adhesion phenotypes before neurulation. Animal cap adhesion assay is as described in (A) and photographed at late gastrula (stage 13) and neurula (stage 18) stages. (C) Rescue of adherens junctions. Whole-mount immunofluorescence of embryos injected is as described in (A). Maximum intensity projections of confocal z stacks are shown. See also Figure S3 and Table S3.

We selected POUV targets (with a >2-fold change in PVD morphants) conserved in both mouse and human, identified as a direct target in at least three studies (N = 32 out of 57), and tested their capacity to rescue PVD2 phenotypes (Figures 3A and 3B). Because E-cadherin appeared a potential mediator of these phenotypes we tested it alongside the transcriptional targets. To evaluate the ability of specific targets to rescue, we scored the number of animal caps that remained intact up to late neurula stage (stages 16–18). The genes tested, their rescue indexes, and known expression patterns or phenotypes are listed in Table S3. We noticed that all direct membrane-associated adhesion regulators, such as E-cadherin, NF-protocadherin, and paraxial protocadherin, had early rescue activity at late gastrula/early neurula stages (stages 13–14), but were unable to sustain ectoderm integrity up to stage 16 and later (Figure 3B). The most effective late-stage rescuers were all transcription factors. Three of these conserved regulators, Xlim5 (Lhx5), Xcad2 (Cdx1), and Xsal1 (Sall1), exhibited rescue activities of PVD2 animal caps approaching 70% or greater. Similar rescue efficiencies were obtained with mouse Oct4 and Xlpou25, whereas Xlpou91 had only partial (early) activity (Figure 3A). Xlim5/Lhx5 is a LIM homeodomain protein highly expressed in the gastrula ectoderm [35] and is closely related to Xlim1, a key conserved regulator of mesendoderm migration. However, Xlim1 was unable to rescue cell adhesion in PVD2 morphant ectoderm (Figure 3A). Xcad2/Cdx1 is a homeobox containing transcription factor involved in anterior-posterior patterning [36]. Xsal1/Sall1 is an ESC transcription factor that regulates differentiation and is known to interact with Nanog and Sox2 [37].

Adherens junctions mediate cell-cell adhesion between adjacent epithelial cells. In *Xenopus* ectoderm they are maintained initially by C-cadherin at early gastrula stages (stage 10.5), and later C-cadherin acts in conjunction with E-cadherin at stage 12 [38]. C- or E-cadherin expression also correlates with increased β-catenin staining in the superficial ectoderm layer [39]. Figure 3C shows that as early as stage 10.5 we observed a substantial reduction in membrane-associated C-cadherin and β-catenin in PVD2 morphant ectoderm. By stage 12, a similar defect was observed in E-cadherin expression and localization. All three of these proteins exhibited a reduced and discontinuous peripheral staining pattern in PVD2 morphants (Figures 3C and S3A). POUV targets that were able to rescue ectoderm integrity (Xcad2/Cdx1 and Xlim5/Lhx5) and Xlpou25 were also able to support membrane localization of these adhesion proteins. Ectopic expression of E-cadherin in POUV morphants (Figures 3C and S3B) restored β-catenin localization, but had no effect on C-cadherin expression (Figure 3C).

We also found that the loss of epithelial morphology in PVD2-depleted ectoderm was accompanied by the acquisition of differential adhesion properties. Unlike the dissociated cells from control animal caps, PVD2 animal explants would not aggregate with other animal caps, but could aggregate with isolated vegetal hemispheres (Figure S3C). Xlim5 was of particular interest because it was previously shown to regulate ectodermal cell adhesion and sorting [34]. Therefore, we tested its effect on PVD2 explant aggregation and found that Xlim5 expression restores the ability of POUV-depleted ectoderm cells to aggregate with control ectoderm.

### Conserved POUV Targets Rescue PVD2 Gastrulation Phenotypes

**Figure 4:**
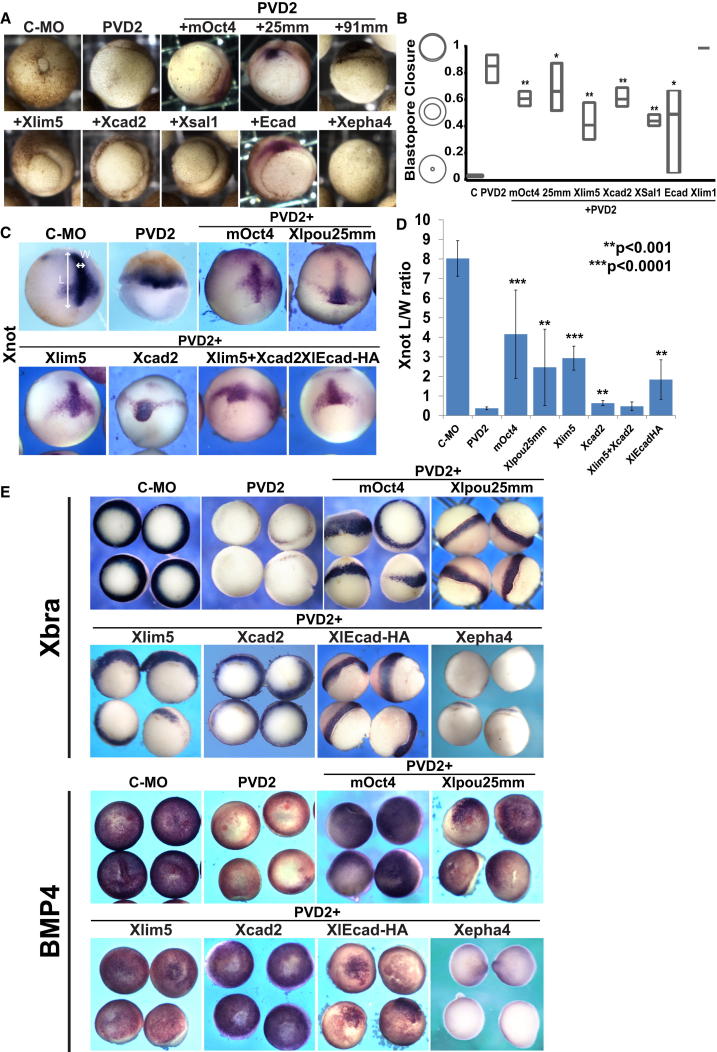
POUV Targets Rescue PVD2 Embryonic Phenotypes (A) Rescue of blastopore closure. Photographs are of whole embryos at stage 12, injected as previously described. (B) Quantification of blastopore rescue. Blastopore closure was estimated at stage 12 using the ratio of blastopore diameter to embryo diameter. The Xlim1 value was arbitrarily set to 1. Box plot shows maximum and minimum values, and the line represents the mean. p values were calculated by nonparametric ANOVA test with multiple comparison corrections (^∗^p < 0.05, ^∗∗^p < 0.001). (C) Rescue of convergent extension defect in PVD2 morphants. In situ hybridization for the dorsal midline marker *Xnot* at stage 12 was used to evaluate convergent extension in PVD2 morphants and rescued embryos [40]. (D) Quantification of convergent extension based on the length/width ratio of *Xnot* (as shown by double arrows on Control-MO). Bars show mean ± SD. p values were calculated as described in (B). ^∗∗^p < 0.001; ^∗∗∗^p < 0.0001. (E) Progenitor state gene expression was rescued by POUV targets. In situ hybridization for *Xbra*, vegetal view, and *Bmp4*, animal view, in PVD2 morphants is shown. See also Figure S4.

PVD2 morphant embryos display a range of morphogenic defects including failures in convergent extension (Figure S1C) and blastopore closure (Figures 4A and 4B). Because there is a close link between adhesion and the morphogenetic movements of gastrulation, we assessed whether conserved POUV targets could rescue these phenotypes. Factors that rescue ectodermal adhesion, in particular, Xlim5/Lhx5 and Xsal1/Sall1, were able to partially rescue blastopore closure (Figures 4A and 4B) and convergent extension as judged by Xnot expression [40] (Figures 4C and 4D). Both phenotypes were also rescued by Xlpou25 and to varying extents by E-cadherin (Figures 4A–4D).

We also tested the capacity of some of the most effective adhesion rescuers to support gastrulation-stage gene expression in the absence of POUV proteins. We found that Xlim5/Lhx5, Xcad2/Cdx1, Xlpou25 or mOct4, and to a limited extent E-cadherin rescued progenitor cell markers such as *Brachyury* and *Bmp4* (Figure 4E).

### Forced E-Cadherin Expression Transiently Blocks ESC Differentiation

**Figure 5:**
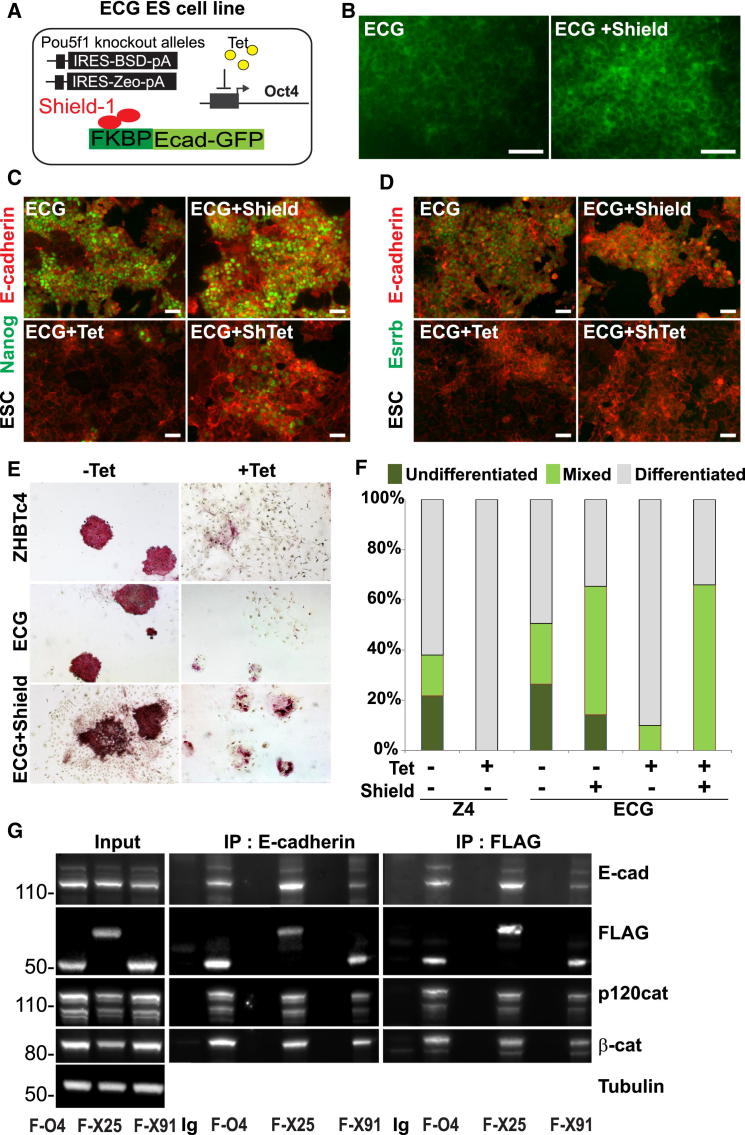
E-Cadherin Expression Partially Blocks ESC Differentiation (A) Schematic of ZHBTc4-derived inducible E-cadherin-GFP (ECG) rescue ESC line. Randomly integrated FKBP-E-cadherin-GFP was constitutively expressed from the CAG promoter. (B) Induction of E-cadherin-GFP with Shield-1. ECG ESCs were cultured under self-renewing conditions with or without Shield and imaged for GFP expression. The scale bar represents 50 μm. (C–D) Induction of E-cadherin in ECG cells supports low-level expression of Nanog (C), but not Essrb (D). Cells were cultured in the presence/absence of Shield and/or tetracycline and then fixed for immunofluorescence. The scale bar represents 50 μm. (E) E-cadherin supported limited AP-positive clonal growth of ESCs in the absence of Oct4. ECG ESCs were plated at clonal density in the presence/absence of Shield and/or tetracycline and colonies stained for tissue-nonspecific alkaline phosphatase (AP; red) after 7 days of growth. (F) Quantitation of clonal growth in ESCs supported by E-cadherin in place of Oct4. Colonies were scored as undifferentiated (dark green), mixed (light green), and differentiated (gray). (G) E-cadherin and POUV proteins interact. Postnuclear membrane fractions were purified from FLAG-tagged POUV-protein-rescued cell lines (see Figure 6) grown in self-renewing conditions. The figure shows immunoblot of coimmunoprecipitations, precipitating with either E-cadherin or the FLAG tag antibody. F-O4, FLAG-Oct4; F-X25, FLAG-Xlpou25; F-X91, FLAG-Xlpou91; Ig, IgG control. See also Figure S5.

We tested the capacity of forced E-cadherin expression to stabilize ESC self-renewal in the absence of Oct4 using a ZHBTc4 ESC line harboring an inducible E-cadherin protein (Figure 5A). An E-cadherin-GFP fusion was rendered unstable by the addition of an FKBP peptide [41] and introduced into ZHBTc4 ESCs. Binding of the synthetic ligand Shield-1 to the FKBP tag blocks degradation of E-cadherin and enables stable expression (Figure 5B). Induction of E-cadherin with Shield-1 upon Oct4 depletion demonstrated that E-cadherin could not block differentiation, but was able to support a population of Nanog-low, Essrb-negative cells (Figures 5C and 5D). The ability of E-cadherin to partially block differentiation was also observed at clonal density, where E-cadherin supported the expansion of partially differentiated AP-positive colonies in the absence of Oct4 (Figures 5E and 5F).

**Figure 6:**
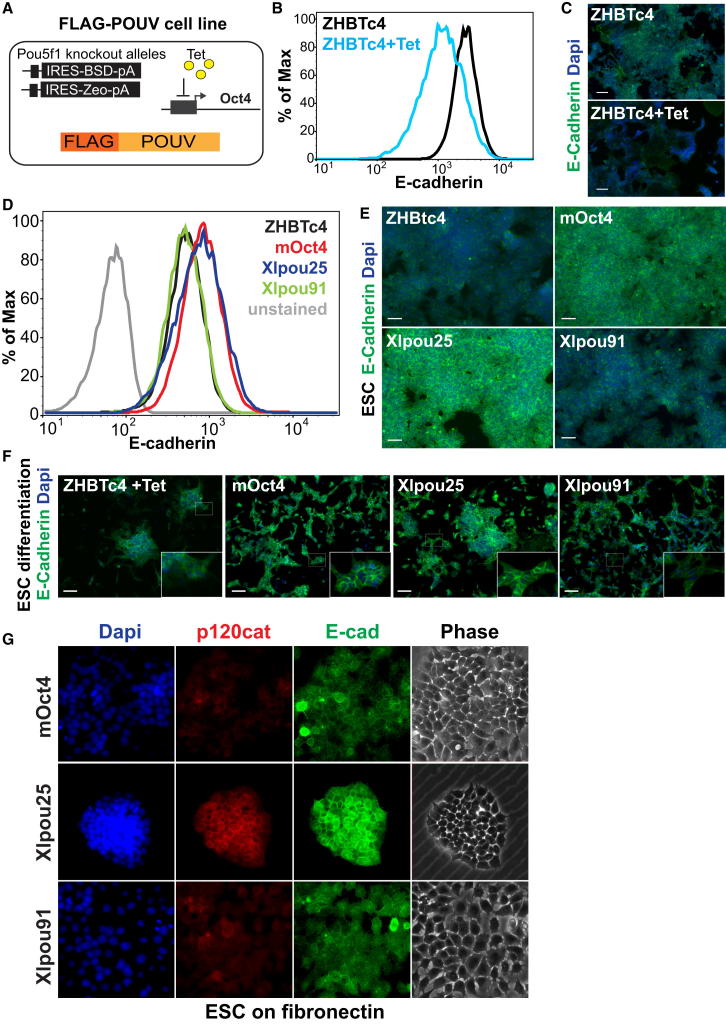
POUV Proteins Support Epithelialization and Antagonize EMT (A) The diagram shows rescue of ZHBTc4 ESCs with different FLAG-tagged POUV proteins. POUV proteins were introduced into these cells in the presence of tetracycline (Tet), and cell lines expressing similar and Oct4-like levels of POUV proteins were selected for further analysis. See also Figure S5A. (B–C) Downregulation of E-cadherin in response to Oct4 depletion in ZHBTc4 ESCs. ZHBTc4 ESCs were plated in self-renewing conditions in either the presence or absence of Tet and assessed by flow cytometry (B) or immunofluorescence (C). The scale bar represents 50 μm. (D–E) POUV- and, particularly, Xlpou25-rescued cell lines express localized E-cadherin. FLAG-POUV cell lines were cultured under self-renewing conditions and assessed by flow cytometry (D) or immunofluorescence (E). The scale bar represents 50 μm. (F) POUV- and, particularly, Xlpou25-rescued cell lines maintain normal epithelial morphology during mesoderm and endoderm differentiation. Immunofluorescence on cultures differentiated for 4 days. The scale bar represents 50 μm. (G) Xlpou25-supported ESCs are particularly resistant to the induction of EMT. ESC lines rescued by the indicated POUV protein were plated on fibronectin to stimulate EMT. Morphology and expression of E-cadherin and p120-catenin were assessed by immunofluorescence microscopy. See also Figure S5.

Thus, both in frog and ESCs, E-cadherin was able to transiently rescue Oct4 depletion, and, therefore, we explored whether Oct4 could stabilize E-cadherin through direct protein-protein interaction. It has also recently been shown that Oct4 can be sequestered at the membrane through an interaction with β-catenin [42], suggesting Oct4 may sustain E-cadherin levels via a direct interaction. We used variants of the ZHBTc4 ESCs in which Oct4 activity had been rescued by FLAG-tagged POUV proteins expressed at levels known to support self-renewal (see Figure 6A; Figure S5A). Figure 5G shows that under conditions designed to detect interactions between membrane proteins we were able to precipitate Oct4 or either *Xenopus* POUV protein with E-cadherin in the same complex together with p120-catenin and β-catenin.

### POUV Proteins Support Adhesion in Mouse ESCs

Because E-cadherin transiently supports adhesion and self-renewal in both ESCs and *Xenopus*, we further explored whether sustained rescue of ESC phenotypes by POUV proteins was associated with epithelial phenotypes. We had previously shown that Xlpou91 was able to support ZHBTc4 ESCs in the absence of Oct4 [9], and it was recently shown that Xlpou91 was also effective in place of Oct4 in the generation of iPSCs [5]. Of the *Xenopus* POUV proteins, Xlpou25 appears to be the major adhesion regulator, although in ESCs it was not as efficient as Xlpou91 at supporting self-renewal. To explore these differences further, we used the cell lines depicted in Figure 6A that expressed similar levels of FLAG-POUV proteins in place of Oct4 (Figure S5A). As a formal test of ESC potency, teratomas were generated from these lines and found to contain embryonal carcinoma cells and partially differentiated tissues from all three germ layers, indicating that these POUV proteins can functionally rescue ESC potency (Figure S5B). Thus, although Xlpou91 was an effective mediator of ESC expansion, both Xlpou25 and Xlpou91 were able to support germ layer specification in teratoma formation.

We next asked how E-cadherin expression in ESCs responded to both Oct4 knockdown and expression of different POUV proteins (Figures 6A–6E). E-cadherin levels were dependent on Oct4 (Figures 6B and 6C), and the expression of different POUV proteins in place of Oct4 supported E-cadherin (Figures 6D and 6E). We found that although both Xlpou25 and Xlpou91 sustained localized E-cadherin expression, Xlpou25 was able to drive higher levels, similar to those achieved by Oct4 (Figures 6D and 6E). When these cells were differentiated toward mesoderm and endoderm, Xlpou25 also better maintained epithelial morphology (Figure 6F), although both Xlpou25 and Xlpou91 were able to support epiblast gene expression (Figure S5C). Finally, we plated these lines onto fibronectin to force E-cadherin downregulation and determine whether constitutive expression of POUV proteins can inhibit epithelial to mesenchymal transition (EMT) induced by fibronectin. Figure 6G shows that Xlpou25, and to a more limited extent Oct4, effectively maintained E-cadherin expression and localization, blocking EMT and supporting epithelial morphology on fibronectin. Under these conditions, Xlpou25 was also able to sustain high levels of p120-catenin (Figure 6G).

## Discussion

The maintenance of pluripotent populations is a conserved facet of development, and as a result pluripotency can be better understood when viewed alongside early embryonic differentiation. We have identified a set of conserved targets of Oct4 and other POUV proteins that links the regulation of cell-cell adhesion and the exit of uncommitted epithelial progenitor cells into differentiation.

In mammalian embryos there are several stem-cell-like populations, including the early preimplantation embryo (naive pluripotency), the later epiblast (primed), and primordial germ cells (PGCs) [1]. Each of these cells can give rise to in vitro lines that perpetuate pluripotency through self-renewing cell divisions. A number of factors expressed in preimplantation development and naive ESCs are also expressed in PGCs. As a result, it is intriguing that Xlpou91, the *Xenopus* POUV protein expressed in the PGCs [43], is best able to rescue ESC self-renewal, suggesting that perhaps naive pluripotency is derived from the regulatory program in the ancestral PGC lineage. Primed pluripotency is more likely to be highly conserved, given the degree to which gastrulation itself is conserved as a process that both requires a continuous pool of progenitors for progressive lineage allocation and is tightly linked to axis formation. In *Xenopus* Xlpou25 is the main regulator of gastrulation-stage primed pluripotency. As a result, it would appear that *Xenopus* has evolved independent networks for the maintenance of naive and primed pluripotency following the gene duplication event that produced three Class V POU proteins. We show that Xlpou25, the most abundant POUV protein at gastrulation, is absolutely required to maintain the integrity of the multipotent ectodermal epithelium and to enable convergent extension. Xlpou25 is also better at supporting epithelial phenotypes in mammalian cells. However, E-cadherin’s capacity to block ESC differentiation and the ability of Xlpou25-supported ESCs to form teratomas imply cell-cell adhesion is important for pluripotency at all levels.

Based on evolutionary conservation we simplified a list of thousands of potential Oct4 targets to fewer than 100 conserved targets. These conserved targets are significantly enriched for regulators of cell adhesion and motility. Thus, we suggest Oct4 preserves epithelial integrity in the epiblast and the induction of EMT in this state may be a key step in committing to differentiation. This idea contrasts with recent observations that the zebrafish POUV protein, DrPou2 (Drpou5f3) [44], controls EGF expression to promote E-cadherin endocytosis. However, DrPou2 is unable to effectively support ESC self-renewal [9, 12] or mediate reprogramming [5], indicating that POUV activity is not well conserved in teleost fish. Moreover, E-cadherin, a key member of adherens junctions, can also replace exogenous Oct4 in reprogramming by supporting mesenchymal to epithelial transition [45]. In differentiation and reprogramming the support of adhesion by E-cadherin is therefore sufficient to transiently substitute for POUV activity, but on its own E-cadherin expression is not sufficient to sustain self-renewal. Sustained maintenance of multipotent progenitors and support of adherens junctions within this population require the activity of the Oct4/POUV. POUV proteins could achieve this in part through the direct E-cadherin-POUV interaction we have shown. However, the ability of transcription factors, such as Lhx5, Sall1, and Cdx1, to rescue POUV phenotypes argues that the activity of the network is sufficient to sustain epithelial integrity and block differentiation. In other contexts these factors have also been associated with adhesion and migration. Xlim5/Lhx5 has a similar adhesion phenotype in *Xenopus* [34] and is a major mediator of ectoderm identity. In mouse there is redundancy in this family, and Lhx5 is specifically required for hippocampal neurogenesis and migration [46]. Although Cdx genes have mostly been implicated in regional identity, Cdx1 regulates cell adhesion through phosphorylation of catenins in human colon carcinoma cells [47]. Finally, Sall1 is also a part of a redundant family of pluripotency regulators related to the *Drosophila* Spalt. In the *Drosophila* nervous system, Spalt regulates the transcription of cytoskeletal and adhesion regulators such as N-cadherin, Fasciclin 2, Fasciclin 3, and the *Drosophila* homolog of β-catenin, Armadillo [48].

The link between adhesion and mediolateral intercalation or ingression has long been associated with the induction of differentiation programs. Our conserved target list features regulators of Nodal and Wnt signaling, pathways associated with gastrulation, epithelial homeostasis, and EMT. Thus, Oct4 does not only suppress differentiation-inducing pathways that promote migration, but also blocks delamination itself. This block of delamination would be essential for normal gastrulation movements and the proper distribution of morphogens within a coherent epithelium. Whether Oct4 regulates pluripotency via adhesion or regulates both in parallel is difficult to resolve from our data set because the effective rescuers are transcription factors. However, the nature of the conserved Oct4 network suggests that the regulation of adhesion and differentiation are inseparable. Thus, although the global effects of modulating adhesion make it difficult to establish a direct functional link between adhesion and pluripotency, it would appear that the ancient role of POUV proteins in triploblastic development is to preserve the competence of cells to both gastrulate and differentiate by retaining them within the uncommitted ectodermal epithelium.

## Experimental Procedures

### *Xenopus* Embryo Microinjection and Manipulation

*Xenopus laevis* embryos were obtained by in vitro fertilization. Microinjection, in situ hybridization, and TUNEL assay were performed as described in [9]. Morpholino oligos (GeneTools) are listed in Table S1.

Immunocytochemistry was performed according to [49], and details are given in the Supplemental Experimental Procedures. XlE-cadherin-HA expression vector was a gift of P. McCrea [50]. Animal cap viability assay was performed as described in [34], using Sytox Green (1 μM; Invitrogen).

### Ectoderm Rescue Assay

In vitro transcribed mRNA (mMachine; Ambion) from I.M.A.G.E. clones was coinjected with morpholino and Texas red dextran (Molecular Probes; Invitrogen). Animal caps were dissected at stage 8 and photographed at stages 13 and 16–18. Rescue activity was evaluated after neurulation (stages 16–18) as the percentage of those retaining their integrity (see Table S3). Opl/Zic1 plasmid was a gift from H. Sive [51].

### Gene Expression Analysis

RT-PCR was performed as described in [6], and the primers are listed in the Supplemental Experimental Procedures.

### Microarray

Agilent *Xenopus* Gene Expression Microarrays 4×44K (AMADID-015066) were hybridized according to the manufacturer’s instructions. Three-way ANOVA was performed as described in [52]. FDR was estimated using the Benjamini-Hochberg method. Further analysis is available in the Supplemental Experimental Procedures.

### Cell Culture and Differentiation

mESCs were cultured as described by [9] and differentiated to mesoderm and endoderm according to [17] and [53]. Stable lines expressing FLAG-POUV or FKBP-E-cadherin-GFP proteins were generated by electroporation of ZHBTc4 ESCs, as described in [9]. EpiSCs were cultured according to [54], and tamoxifen (Sigma) 0.1μM was used to induce recombinase activity.

Immunofluorescence, immunoprecipitation, immunoblotting, flow cytometry, and teratoma assays are all described in the Supplemental Experimental Procedures.

### Ethics Statement

All animal work was conducted in accordance with UK and European legislation and, in particular, according to the regulations described in the Animals (Scientific Procedures) Act of 1986 (UK). All work in this manuscript was authorized by and carried out under Project License 60/3715 issued by the UK Home Office. Genetic modification for the generation of mouse ESC and EpiSC lines was approved by the ethics committees of the University of Edinburgh and the University of Copenhagen.

## Accession Numbers

The Gene Expression Omnibus (GEO) accession number for the microarray data reported in this paper is GSE47578.

## Supplemental Information

Document S1. Supplemental Experimental Procedures, Figures S1–S5, and Tables S1 and S3

Table S2. Gene Expression Data in *Xenopus*, Overlap with Published Data sets and Gene Ontology Analysis, Related to Figure 1
