# Supplementary material for: A Conserved Oct4/POUV-Dependent Network Links Adhesion and Migration to Progenitor Maintenance
Source: Curr Biol. 2013 Nov 18;23(22):2233–44. doi: 10.1016/j.cub.2013.09.048 (PMC4228055; doi:10.1016/j.cub.2013.09.048)
Supplement: Document S1. Supplemental Experimental Procedures, Figures S1–S5, and Tables S1 and S3 [file mmc1.pdf]

Current Biology, Volume *23*

Supplemental Information

A Conserved Oct4/POUV-Dependent

Network Links Adhesion and Migration

to Progenitor Maintenance

Alessandra Livigni, Hanna Peradziryi, Alexei A. Sharov, Gloryn Chia, Fella Hammachi, Rosa Portero Migueles, Woranop Sukparangsi, Salvatore Pernagallo, Mark Bradley, Jennifer Nichols, Minoru S.H. Ko, and Joshua M. Brickman

Livigni2013_Figure S1

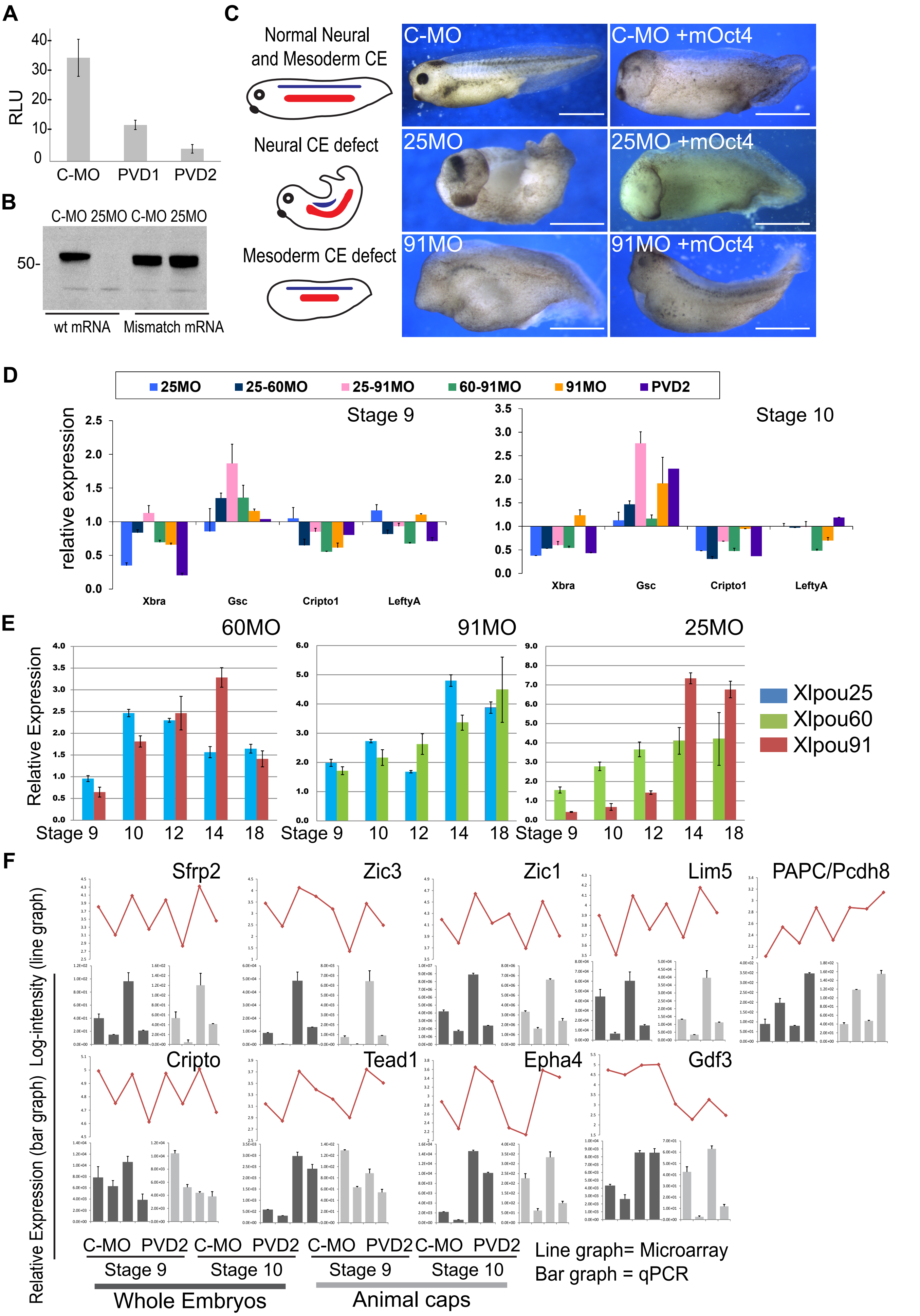

Figure S1 (Refers to Figure 1). POUV Morpholino Phenotypes

(A) PVD2 effectively knocks down POUV activity *in vivo*. Two-cell stage embryos were injected with Control-MO, PVD1, PVD2 and 50pg of an octamer binding motif luciferase reporter. Whole-embryos were assayed for luciferase activity at stage 10.5. (B) Xlpou25MO blocks *in vitro* translation of its target mRNA. Capped mRNA (1 μg) was used for translation in presence of 1 μM MO. The new Xlpou25MO was able to specifically prevent translation of wild-type *Xlpou25* mRNA but could not inhibit translation of *Xlpou25* mismatched mRNA. (C) POUV knockdown phenotypes were rescued by *mOct4* expression. Graphical representation of axial defects in POUV morphants. In normal embryo both neural ectoderm (blue bar) and axial mesoderm (red bar) undergo convergent extension resulting in a straight and elongated axis. Xlpou25 morphants were dorsally kinked suggesting a strong defect in neural convergent extension; they also displayed frequent spina bifida and cyclopia. Xlpou91 depleted embryos displayed a shortened but straight axis indicating a defect in mesodermal extension in conjunction with frequent acephalia. Embryos injected with 40ng of MO and 1ng of *mOct4* mRNA. Photographs at stage 35, scale bar 1mm. (D) qRT-PCR analysis of stage 9 and 10 embryos injected with MO combinations. Expression levels normalized to *Odc* (ornithine decarboxylase) and relative to the Control-MO. Data are shown as mean +/- SD. (E) POUV proteins regulate each other's transcription. POUV transcription in response to single MO (40ng) was analysed by qRT-PCR between blastula and neurula stage (from stage 9 to stage 18). *Xlpou60* was up-regulated early in response to *Xlpou25* knockdown and later during gastrulation in response to *Xlpou91* depletion, however only low levels of expression were observed after gastrulation. Conversely, *Xlpou25* and *Xlpou91* were both up-regulated following *Xlpou60* knockdown. Expression normalised to *Odc* and

relative to Control-MO embryos of the same stage. Data are shown as mean +/- SD. (F) The expression of POUV targets overlapping with the murine and human ChIP datasets was analysed in Xenopus. Bar graphs show qRT-PCR of Control-MO and PVD2 morphant whole embryos and animal caps at stage 9 and 10. Expression levels are normalized to *Odc* (ornithine decarboxylase) and shown as mean +/- SD. Line graphs show the Log-intensity from the microarray samples for comparison. See also Figure 1.

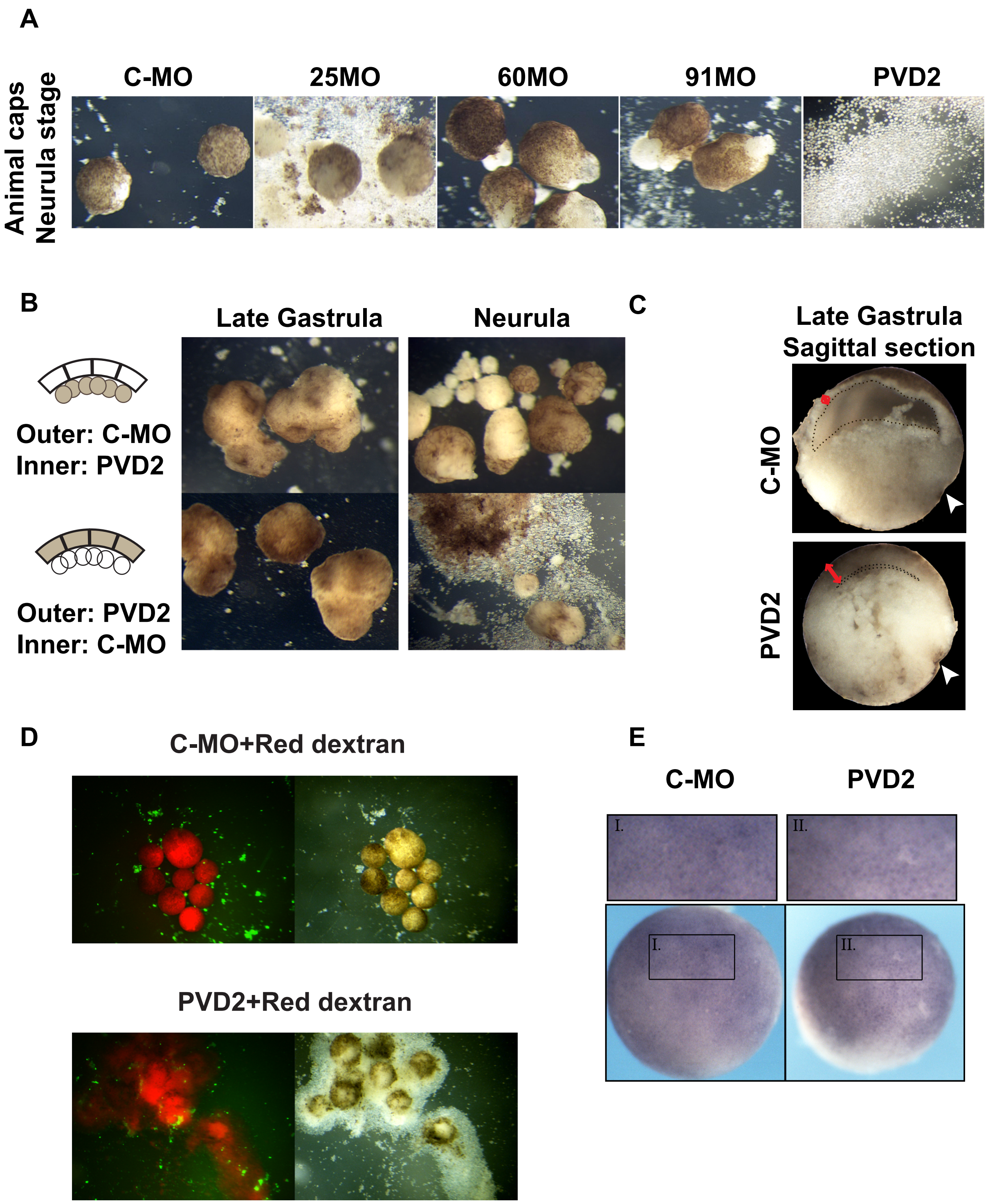
A
Animal caps
Neurula stage
C-MO
25MO
60MO
91MO
PVD2
B
Late Gastrula
Neurula
Outer: C-MO
Inner: PVD2
Outer: PVD2
Inner: C-MO
C
Late Gastrula
Sagittal section
C-MO
PVD2
D
C-MO+Red dextran
PVD2+Red dextran
E
C-MO
PVD2
I.
II.
I.
II.

Figure S2 (Refers to Figure 2). Adhesion Phenotypes in POUV depleted embryos.

(A) The three Xenopus PouV proteins have distinct cell adhesion phenotypes. Animal caps from embryos injected with single MO were dissected at stage 8 and cultured until stage 14 (neurula). Xlpou25 explants exhibited a partial loss of adhesion but PVD2 displayed the most severe adhesion phenotype. (B) Adhesion phenotypes are dependent on the superficial layer of the ectoderm. Animal caps superficial (outer) and inner layer were separated in Calcium-Magnesium free medium (CMFM). Individual layers from control and PVD2 morphant explants were then recombined. PVD2 outer layers were unable to surround the control inner layer cells and completely lost adhesion by neurula stage. However, wild-type outer layers could rescue disaggregation of morphant inner layers (C) POUV depleted embryos displayed defective animal cap epiboly. Ectoderm in PVD2 embryos did not become thinner as in controls (red mark), but appeared as a thick multilayer of densely packed cells. Blastocoel cavity also collapsed in PVD2 embryos (black dotted outline). Photograph showing sagittal section of mid-gastrula embryos, dorsal blastopore lip on the right (white arrow). (D-E) POUV depletion does not increase cell death in animal cap explants or whole-embryos during gastrulation. (D) Animal caps were stained with Sytox-green to identify dead cells and photographed at stage 12.5. TexasRed fluorescent dextran was used as a marker for injection. PVD2 morphants showed the same number of dead cells (green) as the Control-MO. (E) Whole-mount TUNEL staining on stage 10.5 embryos revealed similar extent of cell death between Control-MO and PVD2, photographs showing animal view (ectoderm). Boxed inserts are shown at higher magnification.

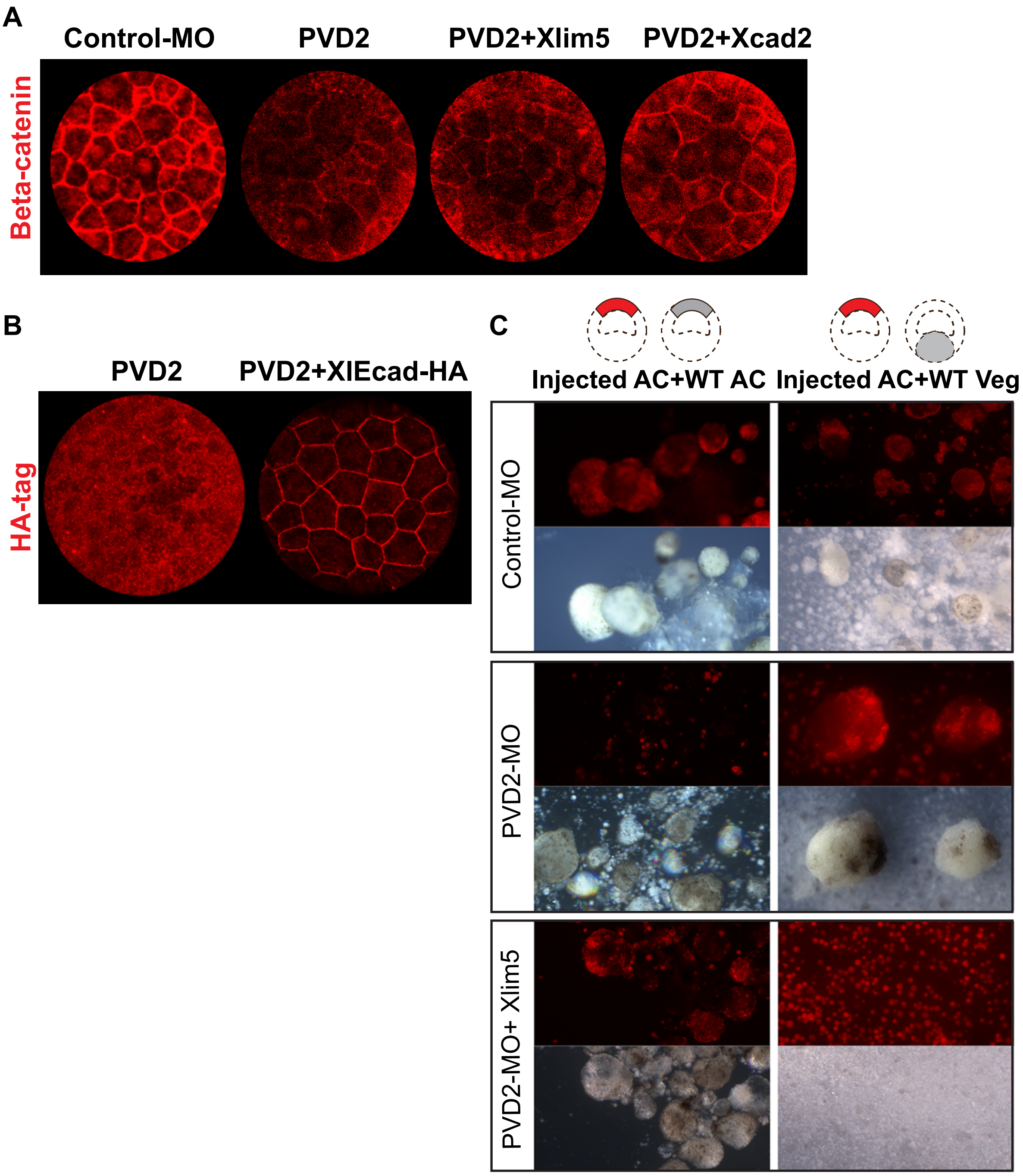
A
Control-MO
PVD2
PVD2+Xlim5
PVD2+Xcad2
Beta-catenin
B
PVD2
PVD2+XlEcad-HA
HA-tag
C
Injected AC+WT AC
Injected AC+WT Veg
Control-MO
PVD2-MO
PVD2-MO+ Xlim5

Figure S3 (Refers to Figure 3). Rescue of Adherens Junctions and Regulation of Differential Adhesion by POUV targets.

(A) POUV targets rescue β-catenin localization in late gastrulae ectoderm. Whole-mount fluorescence of embryos injected with MO and 500pg mRNA of specific POUV targets where indicated. *Xcad2* and *Xlim5* supported localization of β-catenin at cell-cell junctions in late gastrulae (stage 12). Image shows Maximum Intensity Projections (MP) of confocal Z-stacks from stage 12 ectoderm. See also Figure 3. (B) Overexpression of E-cadherin in PVD2 morphants. Whole-mount fluorescence of PVD2 embryos injected with 500pg of mRNA for HA tagged Xenopus *E-cadherin*. Overexpressed E-cadherin localized at the adherens junctions as seen by HA staining. Image shows MP of Z-stacks from stage 12 ectoderm. (C) *Xlim5* regulates differential adhesion in PVD2 morphants. Ectoderm and Vegetal explants were excised at early blastula stage from Control-MO and PVD2 embryos injected with TexasRed dextran, and with or without 500pg of *Xlim5* mRNA. Explants were dissociated in CMFM and recombined. POUV depleted animal explants aggregated poorly with control ectoderm (PVD2 Left panel) and displayed higher affinity for vegetal cells (PVD2 Right panel). *Xlim5* restored the ability of morphant ectoderm cells to aggregate with control ectoderm.

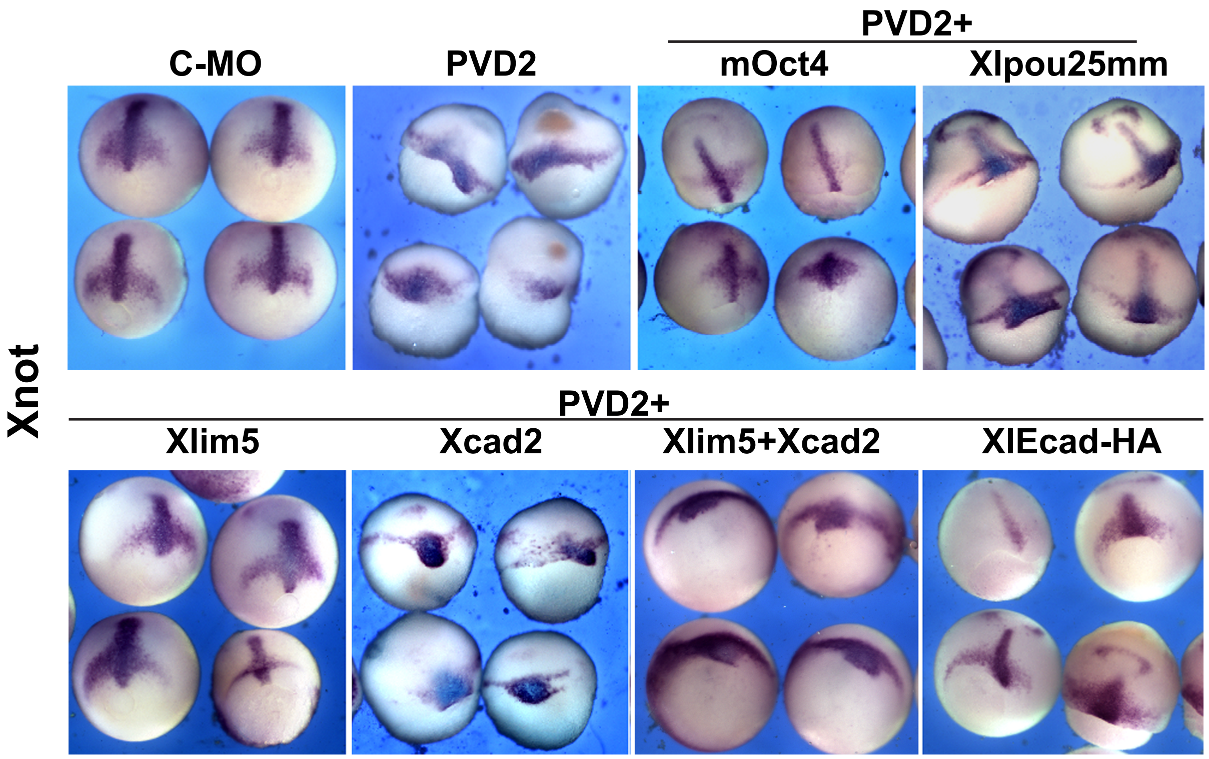
PVD2+
C-MO
PVD2
mOct4
Xlpou25mm
Xnot
PVD2+
Xlim5
Xcad2
Xlim5+Xcad2
XlEcad-HA

Figure S4 (Refers to Figure 4). Rescue of Convergent Extension Defect in PVD2 Embryos.

Embryos were injected with Control-MO or PVD2 (120ng) and 500pg mRNA of the indicated POUV protein or POUV target. Figure shows whole-mount *in situ* hybridisation for the notochord marker *Xnot*, dorsal view. See also Figure 4 for a close up of these embryos.

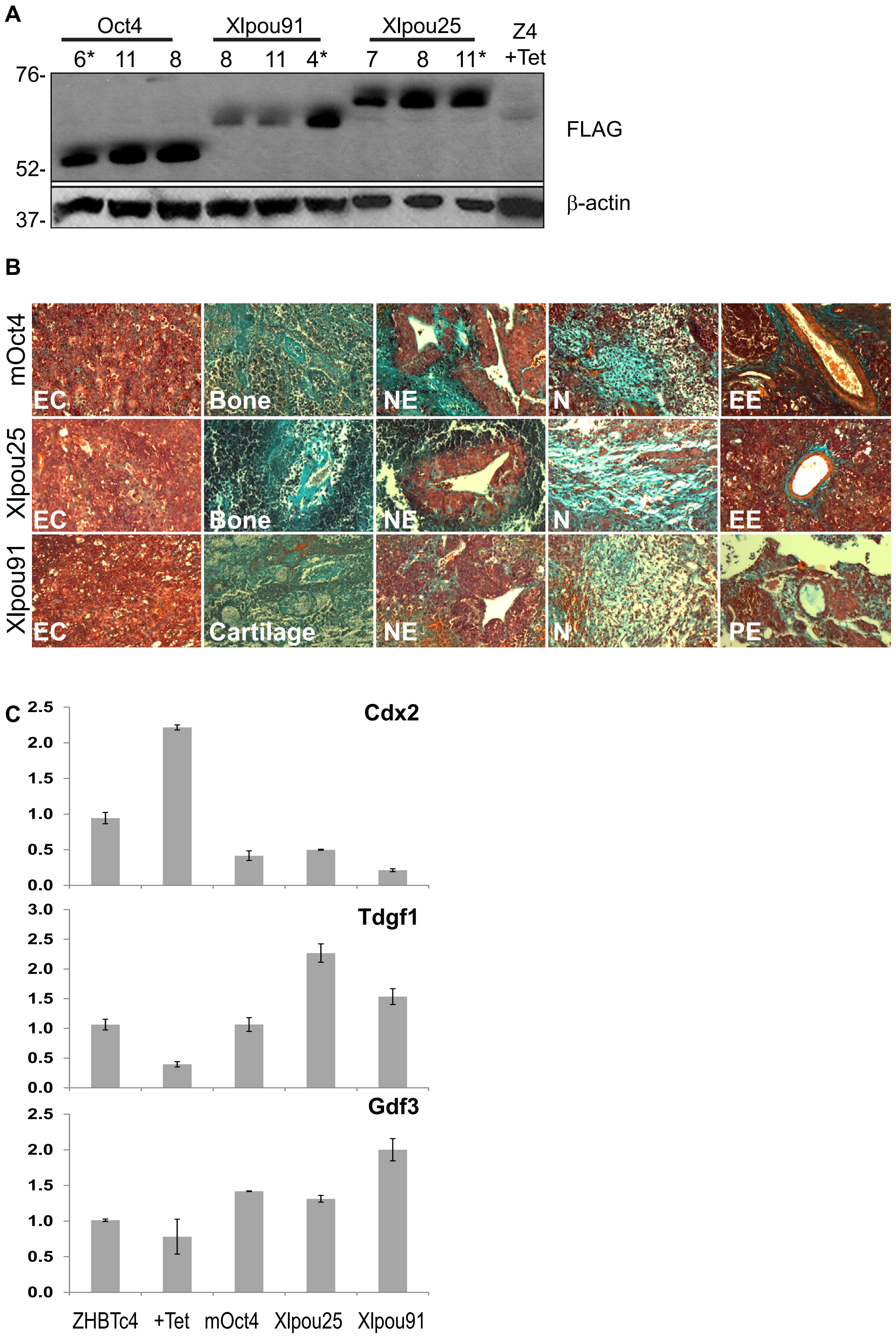
A
Oct4
Xlpou91
Xlpou25
Z4 +Tet
6* 11 8 8 11 4* 7 8 11*
76-
52-
37-
FLAG
β-actin
B
mOct4
Xlpou25
Xlpou91
EC
Bone
NE
N
EE
Cartilage
PE
C
Cdx2
Tdgf1
Gdf3
0.0
0.5
1.0
1.5
2.0
2.5
3.0
ZHBTc4
+Tet
mOct4
Xlpou25
Xlpou91

Figure S5 (Refers to Figure 5 and 6). POUV Rescued Mouse ESC Lines.

(A) Clonal cell lines in which Oct4 was replaced by different FLAG tagged POUV proteins were generated from ZHBTc4 ES cells with FLAG--mOct4, -Xlpou25 and -Xlpou91. All tagged POUV proteins supported ESC self-renewal as previously described. Protein levels are shown by immunoblot. Equal amounts of total protein were loaded based on normalization to β-actin. Three clonal cell lines for each POUV protein are shown in the figure. The experiments in Figure 5, 6 were done with clone 6 (Oct4) clone 11 (Xlpou25) and clone 4 (Xlpou91). These three lines were selected based on their expression of similar levels of the different POUV proteins. (B) ESCs supported by FLAG-POUV are pluripotent. Cells were transferred under the kidney capsule of adult 129/Ola mice. Mice were fed Tetracycline to ensure repression of the Tet-responsive *Oct4* transgene contained in the ZHBTc4 parental line. After 4-5 weeks, tumors were sectioned and subjected to Masson's trichrome staining. Tumors displayed examples of primitive tissues from all three embryonic germ layers. They contained embryonal carcinoma (EC) cells, neuroepithelium (NE), mature neural tissue (N), cartilage, bone, endodermal epithelium (EE), and pulmonary epithelium (PE). (C) FLAG-POUV supported ESCs express epiblast markers during ESC differentiation. Gene expression analysis by qRT-PCR of FLAG-POUV ESC cells following 4 days of differentiation towards mesoderm and endoderm. All three POUV proteins supported the expression of epiblast markers (*Tdgf1*, *Gdf3*) and antagonized *Cdx2* induction in response to Tetracycline. Expression levels are normalized to TBP and shown as mean +/- SD.

Table S1 (Refers to Figure 1). Morpholino Oligos and POUV Phenotypes.

POUV Phenotype scoring							
**Stage 37/39**	**ID**	**N**	**MO (ng)**	**Normal axis length (%)**	**Moderate axis truncation (%)**	**Severe axis truncation (%)**	**Anterior defect (%)***
Not injected	-	112	0	93	7	0	0
Control-MO	0	114	120	92	8	0	0
25MO	1	34	40	6	47	47	76 {17}
60MO	2,4	48	40§	65	21	14	6
60bMO	2	77	40	80	12	8	4
91MO	5	80	40	55	27	18	45 {40}
PVD1	3,4,5	83	120	25	58	17	2
PVD2	1,2,4,5	118	120#	5	26	69	67

Morpholino oligos				
**ID**	**Design**	**Alleles targeted**	**Accession**	**Sequence**
0	Standard Control Oligo from GeneTools			
1	This paper	Xlpou25a<br>Xlpou25b	AJ699165<br>BC079821	AGGGCTGTTGGCTGTACATGGTGTC
2	This paper	Xlpou60b	CA972414	GGCTGTACAATATGGATTGGACCAT
3	Morrison and Brickman 2006	Xlpou25a	AJ699165	ACATGGTGTCCAAGAGCTTGCAGTC
4	Morrison and Brickman 2006	Xlpou60a	X86377	GTACAATATGGGCTGGTCCATCTCC
5	Morrison and Brickman 2006	Xlpou91	M60077	GTAGGTCTGTTGGTTATACATGATC

§ 20ng of each morpholino oligo for 60a (ID 4) and 60b (ID2) were injected

PVD2 120ng= 40ng 25a and b (ID1), 20ng 60b (ID2), 20ng 60a (ID4), 40ng 91(ID5).

*Anterior defect, embryos showing defective eye development including cyclopia, enlarged or reduced cement gland, irrespective of whether these embryos did or didn't demonstrate additional axis extension defects.
In brackets { } percentage of embryos with complete lack of eye and cement gland.

Rescue assay	AC rescue	N	Xenopus ID	Mouse ID	Function	PVD2 MO response (log10)	Oct4-KO mESC (log10)	Early expression Pattern Mouse	Late expression Pattern Mouse	Mouse phenotype
**Blastopore and AC rescue**	78%	69	lim5/Lhx5	Lhx5	TF, AM	-2.13	n.a.	n.a.	cerebellum	nervous system (die-postnatal)
	73%	44	xCAD2	Cdx1	TF, AM	-2.72	n.a.	ICM, PS	limb, posterior mes.	skeleton defects
	67%	40	MGC68418	Sall1	TF, AM	-3.91	n.a.	PS	brain, limb, kidney	kidney agenesis, nervous s. (die-perinatal)
**Partial AC No Blastopore rescue**	63%	25	MGC81567	Tead1	TF, AM	-1.97	n.a.	n.a.	limb, br.arch.	cardiovascular (die-perinatal)
	63%	32	NFPC	Pcdh7	AM	-2.20	n.a.	n.a.	brain	n.a.
	55%	22	Xbra	T	TF, AM	-2.21	-0.178	PS	notochord, tailbud	lack a trunk and tail (die-prenatal)
	≤40%	24	LOC398232	Spry2	AM	-1.98	n.a.	PS	limb, br.arch.	digestive, nervous s. (die-postnatal)
	≤40%	27	Fkh-5	Foxb1	TF	-2.50	n.a.	n.a.	diencephalon	reproductive, nervous s.(die-perinatal)
	≤40%	23	Irx2-A	Irx2	TF	-3.37	n.a.	n.a.	lung, limb	phenotypically normal
	≤40%	22	Gdf3-a	Gdf3	STEM	-2.76	-0.854	Node	notochord	prenatal lethality and resistance to obesity
	≤40%	15	Cr1	Tdgf1	AM	-1.94	-0.853	PS	cerebellum, lung	cardiovascular (die-perinatal)
	≤40%	23	MGC81734	Tgif2	TF, BMP	-1.67	-0.22	n.a.	brain, cord	n.a.
	≤40%	24	C13orf15	Rgc32	AM	-3.88	0.441	n.a.	midbrain	n.a.
	≤40%	15	RTN4	Rtn4	AM	-2.48	n.a.	n.a.	brain	nervous system
**No AC or Blastopore rescue**	≤30%	15	Zic3-A	Zic3	STEM, TF	-11.03	-1.659	PS	brain, lung, liver	cardiovascular, nervous s.(die-perinatal)
	≤30%	20	Foxd3-A	Foxd3	STEM, TF, AM	-1.96	-0.794	n.a.	brain	failure of PS formation and gastrulation
	≤30%	45!	Kit-A	Kit	STEM, AM	-5.02	-0.587	n.a.	b. marrow, melanocytes	hematopoietic, repr. pigment (die-perinatal)
	≤30%	45!	Kitlg	Kitl	AM	-2.24	0.29	widespread	brain, liver	hematopoietic
	≤30%	21	Pag	Epha4	AM	-2.04	-0.575	n.a.	rhombomeres	nervous s., limbs
	≤30%	23	Biklf-A	Klf4	STEM, TF, AM	-3.54	-0.523	E7.5	lung, intestine	digestive (die-perinatal)
	≤30%	18	Sfrp2	Sfrp2	WNT	-7.72	-0.258	E7.5	brain, eye	limbs, reproductive
	≤30%	29	Adamts1	Adamts1	AM	-8.35	-0.177	n.a.	brain,lung, liver	reproductive (die-perinatal)
	≤30%	18	Xror2	Ror2	AM	-3.55	0.242	E7.5 PS	neural crest, br.arch.	limbs, reproductive (die-perinatal)
	≤30%	20	LOC495078	Eomes	TF, AM	-2.46	0.411	PS	brain, limb	fail to implant, and lack trophoectoderm
	≤30%	37	Dkk1-A	Dkk1	WNT	-2.55	1.034	head mes., endo	brain, limb, br.arch.	limb, head defect (die-perinatal)
	≤30%	22	Ppap2bb	Ppap2b	AM	-2.13	n.a.	n.a.	brain, bladder	die E10.5, defect extraemb. vasculogenesis
	≤30%	15	fkh1-A	Foxa2	TF, AM	-2.07	n.a.	PS	gut, notochord	lack node, notochord (die-prenatal)
	≤30%	15	Tcfap2a	Tcfap2a	TF, AM	-1.89	n.a.	n.a.	cerebellum	cardiovascular, nervous s. (die-perinatal)
	≤30%	26	FGF-8b	Fgf8	FGF	-2.33	n.a.	n.a.	neural tube, hindgut	normal at birth, missing brain parts
	≤30%	15	MGC52546	Meis2	TF	-6.02	n.a.	n.a.	brain, eye	n.a.
	≤30%	15	Opl	Zic1	TF	-3.40	n.a.	n.a.	brain, bladder,eye	cerebellar hypoplasia
	≤30%	40	Sip1	Zeb2	TF, AM	-3.09	n.a.	n.a.	brain	die between E9.5 and 10.5

Table S3 (Refers to Figure 3). Rescue Activity and Phenotypes of Conserved POUV Targets.

POUV targets were chosen among the conserved targets down-regulated over 2-fold in our microarray and included in at least 3 ChIP datasets. These targets (n=32) were tested for rescue of PVD2 embryos. Embryos and dissections were performed as described in Figure 3. Rescue activity was estimated by calculating the percentage of caps retaining their integrity divided by the number of animal caps dissected. Caps that retained their integrity were counted when sibling controls reached neurula stage. Data for Oct4 knock-down in mESCs from reference [S1]. Gene information was obtained from www.xenbase.org; www.informatics.jax.org; www.ncbi.nlm.nih.gov.

Abbreviations: AM, Adhesion and Motility regulator; TF, Transcription factor; STEM, regulator of stem cells self-renewal and pluripotency; BMP, WNT, FGF, involved in signalling pathway; PS, Primitive streak; n.a., Not Available.

Supplemental Experimental Procedures

Xlpou25 mismatch.

Five mismatches were introduced in Xlpou25-pCS2 (accession AJ699165) using the following oligos:

D-5-TCA CAGCAA CCT T TCCCAGCCTTCGC

U-5’ ATACATCTGTTCCAAGAGCTTGCAGTCAGA

Xlpou25MO cannot block the translation of the mismatched mRNA (see Figure S1). The protein sequence does not change and this variant was able to rescue POUV depleted embryos.

Imaging of Whole-mount Fluorescence Immunocytochemisty.

After vitelline membrane removal, whole-embryos were fixed for 1h in MEMFA (3.5% Formaldehyde) and stained as described in the main Experimental Procedures section. Imaging was performed positioning embryos on a mesh and using an Apochromat 40×/0.8 W L UV-vis IR immersion objective. Z-stacks were collected using a Leica SP2 upright confocal microscope, 4.37AU β-catenin stage 12 or with a Zeiss LSM710 upright confocal, 1AU for β-catenin at stage 10.5 and the other antibodies. Maximum Intensity Projection (MP) was calculated using Volocity software. For each condition a minimum of 5 embryos were analysed comparing MP from a similar number of Z-stacks. When required stack registration was performed using Fiji.

Animal cap outer and inner layer aggregation assay.

Animal caps were excised at stage 8 and briefly incubated in Calcium magnesium-free medium (CMFM; 88 mM NaCl, 1 mM KCl, 2.4 mM NaHCO3, and 7.5. mM Tris,

pH 7.6) to facilitate the detachment of the inner layers while maintaining the outer layers intact. Control inner cells were scraped off using an eyebrow knife and placed upon the PVD2 morphant outer layers. The opposite combination (PVD2 outer layer and control inner layer) was performed for comparison. Calcium and Magnesium (2mM) were added to allow aggregation. After ~30min the aggregates were transferred in 3/4 NAM (3/4 NAM: 82.5 mM NaCl, 1.5 mM KCl, 0.75 mM Ca(NO3)2, 0.75 mM MgSO4, 75 µM EDTA, 1.5 mM sodium phosphate (pH 7.5), 0.75 mM NaHCO3), incubated with gentle shaking and photographed when siblings embryos reached stage 12 and 14.

Ectoderm and vegetal aggregation assays.

Explants were dissociated in CMFM and outer layer were dissociated with PhoNaK buffer (50mM NaH2PO4, 35mM NaCl, 1mM KCl). Dissociated cells from control and morphant explants were mixed and allowed to aggregate overnight in 3/4 NAM on a Rotator-Nutator shaker. Aggregates were examined with an AZ100 fluorescence microscope (Nikon) and Volocity imaging software (Improvision).

Evaluation of blastopore closure and convergent extension.

Blastopore closure. Embryos were fixed or imaged live when Control-MO reached stage 12. Measurements were performed on collected photographs using Fiji. The ratio of blastopore diameter to embryo diameter was calculated for 15-20 embryos from 2 independent experiments.

Calculation of Xnot length to width ratio: Embryos were subjected to *in situ* hybridisation for Xnot, bleached and photographed in dorsal view, aligning them on a mesh. Length and width of Xnot domain was measured using Fiji for 10-15 embryos

from 2 independent experiments.

Microarray analysis.

Xenopus embryos were injected with 120ng of Control-MO or PVD2. Animal caps were excised at early blastula stage (st.8) and cultured with intact sibling embryos at the same temperature for staging. Explants were cultured until sibling embryos reached late blastula (st.9) and early gastrula (st.10) stage. At both stages RNA was prepared from 3 whole embryos or 20 animal caps using an Rneasy Mini kit (Qiagen). RNA quality check was performed using an Agilent 2100 Bioanalyser and only samples with a RNA integrity number (RIN) higher than 8 were used for cRNA synthesis and labelling. Two independent experiments were used as biological replicates. As reference we used a pool of RNA from wild type Xenopus embryos at the following stages: 2, 4, 6, 8, 9, 10, 11, 12, 15, 18, 20. Labelled samples (Cy3-CTP) and reference (Cy5-CTP) were prepared from 2.5µg of total RNA using Agilent's Quick Amp Labelling Kit. Labelled cRNA were purified using an Rneasy Mini kit (Qiagen) and hybridized to the Agilent Xenopus Gene Expression Microarray (V1) 4x44K AMADID-015066. Arrays were hybridised according to manufacturer's protocol (Two-Color Microarray-Based Gene Expression Analysis Protocol, Version 5.0.1). Slides were scanned with an Agilent DNA Microarray Scanner (model G2505-64120) at 100% PMT for Cy3 channel and 10% PMT for Cy5 channel with a scan resolution of 5µm. Quality controls and data extraction were performed using Agilent Feature extraction software v9.5.3.1. Probe intensity for each sample was normalized to the reference and log transformed. Signal intensity (Cy3) was log-transformed and normalized using reference intensity (Cy5). Three-way ANOVA was performed as described in reference [S2] with factors: (a) PVD2 morphant vs.

control, (b) stage 10 vs. 9, and (c) animal caps vs. whole embryo. Error variance was adjusted by taking the maximum of the actual error variance for each probe and the average error variance for probes with the same signal intensity in a sliding window of 500 genes (default method in the NIA Array Analysis software http://lgsun.grc.nia.nih.gov/ANOVA/). False Discovery Rate (FDR) was estimated using the Benjamini-Hochberg method. Published datasets were analysed using GPAT [S3], Gene Ontology enrichement was performed using GOEAST [S4].

Dataset analysis

Lists of Oct4 targets identified by ChIP-seq or ChIP-on-ChIP studies were obtained from published datasets. When necessary we performed an independent re-analysis assigning peaks with GPAT (http://bips.u-strasbg.fr/GPAT/). Datasets were compared using official GeneSymbols, human identifiers were converted to their mouse homologs. The identifiers were updated to the most recent nomenclature using the MGI website (www.informatics.jax.org). P-values for list overlap were calculated by hyper-geometric distribution. The cumulative probability (P) of observing an intersection of at least X targets (x=123 mouse or 81 human targets) between our list and the examined datasets is calculated by the inverse cumulative hyper-geometric distribution P(X>=x) = 1- hyper-geometric distribution (N, m, k). Where N is total number of genes in mouse genome (N=25436); m, the number of Oct4 targets occurring in each ChIP dataset (mouse m=9486 and human m=4649); k, number of genes sampled (k=201 in our dataset).

Gene Ontology enrichment was performed using GOEAST (Gene Ontology Enrichment Analysis Software Toolkit http://omicslab.genetics.ac.cn/GOEAST/. Fold

enrichment for each GO class was calculated by dividing the number of genes observed in our list (frequency in our dataset) by the number of genes observed in the *M.musculus* genome (frequency in mouse).

Teratoma assay

FLAG-POUV supported ZHBTc4 ESCs were cultured in ESC media with Tetracycline (1µg/ml) . Cells from three cell lines (Oct4 clone 6, Xlpou25 clone 11, Xlpou91 clone 4) were transplanted under the kidney capsule of 8 week old male 129/Ola mice. Mice were fed water containing Tetracycline 2g/L and sucrose 5g/L, in order to silence the Tet-suppressible *Oct4* transgene in ZHBTc4 cells. Mice were culled when an obvious sign of tumour formation was observed or after approximately 4-5 weeks. Tumours formed in all of the mice transplanted with these ESC lines and the tumours were large and either smooth surfaced or moderately cystic (size between 15x10mm and 25x20mm). This tumour morphology is typical of mouse ES cell derived tumours. Tumours were washed in PBS, fixed in PFA, mounted in paraffin. Sections (7µm) were stained using Masson's staining (described below).

Massons staining:

• Xylene 5 mins
• 100% IMS 5 mins
• 90% IMS 5 mins
• 70% IMS 5 mins
• 50% IMS 5 mins
• 30% IMS 5 mins
• H20 5 mins
• Alcian blue 20 mins
• H20 rinse
• Haematoxylin 3 mins
• H20 rinse
• Xylidine red 4 mins

• H20 rinse
• Phosphomolybdic Acid 5 mins
• H20 rinse
• Light green 4-5 seconds
• 70% IMS 3 seconds
• 90%IMZ 1-2 sec
• 100% IMS 5 mins
• Xylene 5 mins
• DPX mount

Co-Immunoprecipitation

Post-nuclear membrane enriched fractions were prepared as described in reference [S5]. Briefly, cells were subjected to swelling in 10mM Tris pH8.0 with Complete Protease Inhibitors (Roche) for 10 minutes and homogenized by syringe (10-20 strokes, needle gauge 23), while monitoring by phase microscopy. Nuclei were removed through the addition of 1 vol of 300mM NaCl and sucrose (20%), followed by centrifugation at 600g for 5 minutes (repeated twice). CHAPS detergent (0.1%) was added to the post-nuclear fractions before BCA protein assay (Biorad). Equal amounts of post-nuclear membrane fractions were subjected to pre-clearing by incubation for 30min with Dynabeads (Invitrogen) pre-blocked with 0.5% BSA. Beads were removed using a magnetic rack. Antibodies for immuno-precipitation were added (1µg for 200 µg of extract) and incubated 4hours or O/N at 4ºC. Immunocomplexes were recovered by incubation with G-protein couples Dynabeads for 1h, washed (3x15min) using PBS 1x with 0.1%CHAPS and 0.1%TritonX100 and resuspended in Laemmli buffer. Immunoprecipitated fractions were resolved on 4-12% Bis-Tris gels (Invitrogen) with MOPS buffer.

Immunoblotting

Whole cellular extracts (Figure S5) were prepared with RIPA lysis buffer (Thermo scientific), Complete Protease Inhibitors (Roche) and 0.5 mM phenylmethylsulfonyl fluoride were added. Extracts were treated with DNA nuclease Benzonase (70664-3, Novagen) for 1 h at 4°C. Protein concentration was quantified using BCA assay (Biorad). Equal amounts of protein (~10 µg) were resolved by SDS-PAGE on NuPage-Novex 10% Bis-Tris gels (NP0302, Invitrogen) and transferred to Hybond ECL membrane (GE Healthcare). Immunoblot analysis was performed with the antibodies listed in the Antibodies Table at the end of this section and HRP conjugated secondary antibodies (Thermo). Chemiluminescent (ECL) signals were quantified by scanning densitometry using ImageJ software. When Alexa-fluor conjugated secondary antibodies were used detection was performed with Chemidoc MP (Biorad).

Immunofluorescence

Cells cultured in adherent monolayer were gently rinsed with phosphate-buffered saline (PBS) and fixed in 3.7% formaldehyde for 15 min at room temperature, then incubated 5min with 0.1M Glycine (pH 7.4). After permeabilization with 0.5% Triton X-100 in PBS for 5 min, the cells were incubated in blocking buffer (PBS 1×,0.1 mg/ml bovine serum albumin, 5% serum of the 2nd antibody species) for 1 h at room temperature. Primary antibodies are listed in the Antibody Table at the end of this section. Alexa Fluor-conjugated secondary antibodies (Invitrogen) were used. Nuclei were stained with DAPI (0.1µg/ml). F-actin was stained using Rhodamine-conjugated phalloidin (1/100; Biotium, cat.00027). Imaging was performed with a Zeiss Axio Observer fluorescence microscope and Axiovision software.

Flow Cytometry

Flow cytometry was used to evaluate E-Cadherin expression in ESC and EpiSC. Cells were collected using Tryple dissociation buffer (Invitrogen); single cells suspension was achieved by gentle pipetting. Following PBS washes cells were resuspended in flow cytometry buffer (1xPBS and 2% FCS) with eFluor 660-conjugated E-cadherin antibody (0.6µg/ml DECMA1 cat. 50-3249 Ebioscience). Cells were washed three times in PBS and resuspended in flow cytometry buffer with DAPI (0.1µg/ml) for the exclusion of dead cells. Samples were analysed on a Fortessa flow cytometer (BD Biosciences). Data was analysed using the FlowJo (TreeStar).

Antibody Table

Antibodies	Cat#	Immunofluorescence	Immunoblot
Oct4 (C10)	Santa Cruz, sc-5279	1/200	1/1000
FLAG	Sigma, F3165	-	1/2000
Tubulin	Sigma, T2200	-	1/5000
E-cadherin	R&D, AF748	1/200	1/1000
p120catenin	BD, 610134	1/250	1/1000
β-catenin	BD, 610154	-	1/1000
Nanog	Ebioscience,14-5761	1/300	-
Esrrb	Gift, I. Chambers lab	1/300	-
**Antibodies used for Xenopus whole-mount fluorescence**			
XlE-cadherin	DSHB, 5D3	1/50	-
C-cadherin	DSHB, 6B6	1/50	-
HA	Cell signalling, 2367	1/50	-
β-catenin	Sigma, c2206	1/50	-

List of the IMAGE Clones used in the Rescue Assays.

Xenopus symbol	Mouse homolog	IMAGE ID
Biklf-A	Klf4	7980258
Cr1	Tdgf1	8330555
Dkk1-A	Dkk1	9040984
fkh1-A	Foxa2	6863181
Fkh-5	Foxb1	8318684
Foxd3-A	Foxd3	8321039
Irx2-A	Irx2	5516094
Kit-A	Kit	4030854
Kitlg	Kitl	4724973
Meis2	Meis2	5571486
NFPC	Pcdh7	8920652
Ppap2bb	Ppap2b	7010465
Rgcc	1190002H23Rik (Rgc32)	7010574
Rtn4	Rtn4	8077505
Sfrp2	Sfrp2	5571003
Sip1	Zeb2	7019052
Tcfap2a	Tcfap2a	4202568
Xadamts1	Adamts1	6863564
Xbra	T	3402478
Xcad2	Cdx1	8319613
Xepha4	EphA4	5537382
Xlim5	Lhx5	6865745
Xror2	Ror2	6989153
Xsal1	Sall1	6862463
Xsprouty2	Spry2	5513422
Xtead1	Tead1	6863098
Xtgif2	Tgif2	6865523
Zic3-A	Zic3	6864781

List of qPCR primers used with Roche LightCycler 480.

Gene	Probe	Ta	Forward	Reverse
M. musculus				
Pou5f1	SYBR	58	GGCGTTCTCTTTGGAAAGGTGTTC	CTCGAACCACATCCTTCTCT
E-Cad	SYBR	58	ATCCTCGCCCTGCTGATT	ACCACCGTTCTCCTCCGTA
Tdgf1	SYBR	58	TTTTACGAGCCGTCGAAGAT	AATTCAAACGCACTGGAAATG
TBra	SYBR	60	GTGACTGCCTACCAGAATGA	ATTGTCCGCATAGGTTGGAG
Mixl1	SYBR	60	AGTTGCTGGAGCTCGTCTTC	AGGGCAATGGAGGAAAACTC
Tbp	SYBR	58	GGGGAGCTGTGATGTGAAGT	CCAGGAAATAATTCTGGCTCA
Eomes	SYBR	58	ACCGGCACCAAACTGAGA	AAGCTCAAGAAAGGAAACATG
Cdx2	SYBR	58	GGAAGCCAAGTGAAAACCAG	CTTGGCTCTGCGGTTCTG
Gdf3	UPL(7)	60	TGTTCGTGGGAACCTGCT	GCCATCTTGGAAAGGTTTCTG
Fgf8	UPL(16)	60	GCTGTTGCACTTGCTGGTT	ATGCTGTGTAAAATTAGGTGAGGA
Snail	UPL(71)	60	GTCTGCACGACCTGTGGAA	CAGGAGAATGGCTTCTCACC
Lefty1	UPL(76)	60	ACTCAGTATGTGGCCCTGCTA	AACCTGCCTGCCACCTCT
Cerl1	SYBR	58	GACTGTGCCCTTCAACCAG	AGCAGTGGGAGCAGAAGC
Sox17	SYBR	60	CACAACGCAGAGCTAAGCAA	CACAACGCAGAGCTAAGCAA
X.laevis				
Odc	SYBR	55	GCCATTGTGAAGACCTCTCTCCATTC	TTCGGGTGATTCCTTGCCAC
Xlpou25	SYBR	55	CGGAGAGTCTGGAGTTCAGG	GGGTTCTGGGGACTCTTCTC
Xlpou60	SYBR	55	AGTTTGCCAAGGAGCTGAAA	CTGCTCCAATAGGGGTTTGA
Xlpou91	SYBR	55	TAGTGATGGGCTGAGCAGTG	GGTGGTCTGGCTGAATGTTT
Bmp4	SYBR	55	AGCCCAGTAAGGATGTGGTG	GCTGCTGAGGTTGAACACAA
Xbra	SYBR	55	TTCTGAAGGTGAGCATGTCG	GTTTGACTTTGCTAAAAGAGACAGG
Gsc	SYBR	55	CACACACAAGTCGCAGAGTAT	ATGTGTGGGGGAGAAAAATAA
Xom	SYBR	55	TGAGACTTGGGCACTGTCTG	CCTCTGTTGAATGGCTTGCT
Fgf8	SYBR	55	CTGGTGACCGACCAACTGAG	ACCAGCCTTCGTACTTGACA
Xlim5	SYBR	55	TGTCTAACAATAGTGGCTTTAGTGGT	TGGCTTCATTAATGTCAAGGTTC
Zic1	SYBR	55	TCCGTTACATGAGGCAGCC	TTGTTGCACGACTTTTTGGG
Tdgf1	SYBR	55	TGATTGTGCAGCAATGTAAGTG	TGATATCACCGCTCCAAGATT
Sfrp2	SYBR	55	GTGATGGGACAAAAGCTTGG	TTTCTGCCACCGCTTGAC
Gdf3	SYBR	55	AGATTGTACATTGACTTCAAGGATGT	GTAACCACGGGGTGCAAT
Zic3	SYBR	55	CAACAGTGAGGAACCTTCCA	GGGCTTTGTTAGTCTGTAGC
Adamts1	SYBR	55	TTTGGAGACGAGTCCAGACAT	GCACCACAGGGTCGTACA
Epha4	SYBR	55	TCTGACTTTGGCATGTCTCG	CCTGGTTGTGTAGGCTGCTT
Tead1	SYBR	55	CCTGCTGTACCAGGTTATAATGC	AGGCCCAGAAGGAATAGGG
PAPC	SYBR	55	CCGTGCGCTACAGGACTTAT	CTGCCAAAGTCCCTATCACTG

Supplemental References

S1. Sharov, A.A., Masui, S., Sharova, L.V., Piao, Y., Aiba, K., Matoba, R., Xin, L., Niwa, H., and Ko, M.S. (2008). Identification of Pou5f1, Sox2, and Nanog downstream target genes with statistical confidence by applying a novel algorithm to time course microarray and genome-wide chromatin immunoprecipitation data. BMC Genomics *9*, 269.

S2. Sharov, A.A., Dudekula, D.B., and Ko, M.S. (2005). A web-based tool for principal component and significance analysis of microarray data. Bioinformatics *21*, 2548-2549.

S3. Krebs, A., Frontini, M., and Tora, L. (2008). GPAT: retrieval of genomic annotation from large genomic position datasets. BMC Bioinformatics *9*, 533.

S4. Zheng, Q., and Wang, X.J. (2008). GOEAST: a web-based software toolkit for Gene Ontology enrichment analysis. Nucleic Acids Res *36*, W358-363.

S5. Livigni, A., Scorziello, A., Agnese, S., Adornetto, A., Carlucci, A., Garbi, C., Castaldo, I., Annunziato, L., Avvedimento, E.V., and Feliciello, A. (2006). Mitochondrial AKAP121 links cAMP and src signaling to oxidative metabolism. Mol Biol Cell *17*, 263-271.
